# Shared and unique patterns of phenotypic diversification along a stream gradient in two congeneric species

**DOI:** 10.1038/srep38971

**Published:** 2016-12-16

**Authors:** Jonas Jourdan, Sarah T. Krause, V. Max Lazar, Claudia Zimmer, Carolin Sommer-Trembo, Lenin Arias-Rodriguez, Sebastian Klaus, Rüdiger Riesch, Martin Plath

**Affiliations:** 1 College of Animal Science and Technology, Northwest A&F University, Yangling, Shaanxi 712100, P.R. China; 2 Goethe University of Frankfurt, Department of Ecology and Evolution, Max-von-Laue-Straße 13, D-60438 Frankfurt am Main, Germany; 3 Department of River Ecology and Conservation, Senckenberg Research Institute and Natural History Museum Frankfurt, Gelnhausen, Germany; 4 División Académica de Ciencias Biológicas, Universidad Juárez Autónoma de Tabasco (UJAT), C.P. 86150 Villahermosa, Tabasco, México; 5 School of Biological Sciences, Royal Holloway University of London, Egham, Surrey, TW20 0EX, UK

## Abstract

Stream ecosystems show gradual variation of various selection factors, which can result in a zonation of species distributions and gradient evolution of morphological and life-history traits within species. Identifying the selective agents underlying such phenotypic evolution is challenging as different species could show shared and/or unique (species-specific) responses to components of the river gradient. We studied a stream gradient inhabited by two mosquitofishes (genus *Gambusia*) in the Río Grijalva basin in southern Mexico and found a patchy distribution pattern of both congeners along a stretch of 100 km, whereby one species was usually dominant at a given site. We uncovered both shared and unique patterns of diversification: some components of the stream gradient, including differences in piscine predation pressure, drove shared patterns of phenotypic divergence, especially in females. Other components of the gradient, particularly abiotic factors (max. annual temperature and temperature range) resulted in unique patterns of divergence, especially in males. Our study highlights the complexity of selective regimes in stream ecosystems. It exemplifies that even closely related, congeneric species can respond in unique ways to the same components of the river gradient and shows how both sexes can exhibit quite different patterns of divergence in multivariate phenotypic character suites.

Environmental gradients provide a unique opportunity to study natural selection^1^. They allow investigating whether and how gradual variation in ecologically-based selection affects adaptive phenotypic differentiation^2^. Evidence for adaptive diversification along environmental gradients stems from studies of latitudinal^3^,^4^,^5^ and altitudinal (i.e., thermal) gradients^6^, as well as gradients formed by environmental stressors like salinity^7^,^8^ or acidification^9^,^10^. A widespread environmental gradient is found in stream ecosystems, in which various abiotic and biotic selection factors vary systematically from source regions over smaller tributaries to slow-flowing lowland rivers^11^,^12^,^13^. Low-diversity headwater communities are often subjected to strongly variable abiotic conditions and recurrent catastrophic flooding (e.g., after snow melt), while abiotic conditions are more stable in downstream river portions, where multiple tributaries interconnect to form an extensive wetland system and ecological communities become more speciose^11^,^14^,^15^,^16^.

Evolutionary diversification along repeated stream gradients has been particularly well investigated in northern Trinidad, where populations of the livebearing freshwater fish *Poecilia reticulata* (the guppy; family Poeciliidae) occur from the mountainous source regions to lowland portions of river systems. Fast-flowing lower-order creeks are characterized by dense canopy cover, low algal primary production and thus, low food availability for the algivorous guppies^17^ (but see also^18^). This results in low population densities of guppies and an absence of larger predatory fishes^19^,^20^,^21^,^22^. Lowland rivers are slow-flowing, accumulate more nutrients, have higher photosynthetic primary production and thus, higher densities of guppies, and harbour an array of predatory species^17^,^20^,^21^,^22^,^23^,^24^. Guppies show a repeated and predictable pattern of life-history divergence along this gradient, which was mainly interpreted as a consequence of differences in predation risk: under high predation (i.e., increased extrinsic mortality rates), guppy females produce more, but smaller offspring and allocate more resources to reproduction^21^,^23^,^25^, while males mature at an earlier age and develop less conspicuous secondary sexual ornamentation^26^,^27^. Several studies investigated guppy populations that are separated by waterfalls (allowing for a rather clear distinction between low- and high-predation habitats^21^,^23^), but similar patterns of phenotypic differentiation were also found along a continuous gradient of predation^28^.

River systems comprise complex environmental gradients, and so it often remains unclear which components of the river gradient drive patterns of phenotypic divergence in fishes. Circumstantial evidence for the role of stream velocity governing morphological evolution stems from studies on the effects of impoundments (dams), where water reservoirs reduce flow velocity and thus create artificial ‘downstream conditions’^29^,^30^,^31^. Physical characteristics of reservoirs (i.e., altered flow characteristics) appear to drive changes in a set of morphological traits: fish are usually deeper-bodied and have smaller heads in reservoirs^29^,^31^. This likely increases manoeuvrability when feeding on prey suspended in the water column, while more streamlined body contours increase locomotor performance in lotic environments^32^. Moreover, morphological diversification in fishes is linked to predation regimes^33^,^34^,^35^. Specifically, fish are predicted to evolve an enlarged caudal region (body region stretching from the dorsal and anal fins to the caudal fin base) and to have smaller anterior body ⁄ head regions under high predation pressure, which improves predator escape performance through an increased burst speed^33^,^34^,^36^.

Studying gradient evolution—gradual phenotypic divergence in multiple character suites, including evolved differences and adaptive phenotypic plasticity—becomes possible when populations of the same species have adapted to divergent conditions along environmental gradients. In reality, however, different species tend to compete along those gradients, and site-specific competitive advantages of ecologically similar taxa (i.e., competitive exclusion) structure local species compositions^14^,^37^,^38^. Various abiotic factors are known to determine the distribution limits of species along environmental gradients^39^,^40^. In stream ecosystems, salinity, water velocity, temperature regimes, and dissolved oxygen are of particular importance^11^,^14^. Other studies found interactions between biotic and abiotic variables to predict species distributions along environmental gradients^37^,^41^, as exemplified by the study of Torres-Dowdall *et al*.^42^, who examined ecological factors explaining the parapatric distribution of the congeners *P. reticulata* and *P. picta* in the lowlands of Trinidad. It appears as if the distribution of *P. reticulata* is limited by an abiotic factor (increasing salinity), whereas that of *P. picta* is limited by a biotic interaction (interspecific competition with *P. reticulata*)^42^.

In our present study, we examined patterns of phenotypic (i.e., morphological and life-history) diversification along the river gradient of the southern Mexican Río Grijalva^43^,^44^. We focused on members of mosquitofishes (genus *Gambusia*; Poeciliidae^45^,^46^,^47^), a widespread group of freshwater fishes in Central and North America^45^,^46^. In the Grijalva basin, three species of mosquitofishes have been described: widemouth gambusia (*G. eurystoma*) are endemic to hydrogen sulphide-rich spring complexes at the Baños del Azufre^48^,^49^,^50^, while two other species occur throughout the Río Grijalva basin: the teardrop mosquitofish (*G. sexradiata*; Fig. 1A,B) and the Yucatan gambusia (*G. yucatana*; Fig. 1C,D)^43^. Even though both species can reach high local abundances, only few studies reported aspects of their ecology, including trophic ecology^51^,^52^,^53^ and microhabitat preferences^53^, as well as morphological characteristics within and among species^45^,^54^,^55^. Previous reports on Mexican and Belizean ichthyofauna suggest that *G. yucatana* may occur more in coastal waters; however, the species is occasionally also found in inland waters^55^,^56^. The opposite pattern was reported for *G. sexradiata*^45^,^55^,^57^. This distribution pattern is reflected by different salinity tolerances: *G. sexradiata* exhibits a lower tolerance to sea water compared to *G. yucatana*^57^, while *G. yucatana* is even known from some marine habitats^55^,^57^. However, both species co-occur at some sites^55^,^56^, raising the question of what (additional) factors predict their distribution.

The co-occurrence of two morphologically similar congeners in the Grijalva basin prompted a set of questions regarding both their distribution patterns and patterns of phenotypic divergence along the stream gradient (Fig. 2). Even though the existing literature suggests otherwise (refs 55 and 56; see above), both species might co-occur at least in low frequencies along the entire river gradient (Fig. 2A–C). Alternatively, one species might occupy the up-, and the other the downstream portions of the stream. Given that hybridization has been demonstrated to occur even between more distantly related poeciilids^58^,^59^, this type of distribution could result in a hybrid zone where both distributions meet (Fig. 2D–F). Finally, only some components of the river gradient might predict species distribution patterns, leading to a patchy distribution along the river gradient (Fig. 2G–I). In all three cases, phenotypic differences between and within species could be shaped by the following processes: (1) differences could reflect a phylogenetic signal that is independent of the river gradient (statistically, this would result in a significant main effect of the factor ‘species’ in multivariate analyses of variance; see Fig. 2A,D,G), (2) both species could show the same (shared) pattern of gradient evolution (i.e., a significant effect of the covariate ‘environmental gradient’; Fig. 2B,E,H), or (3) both species could respond differently to components of the river gradient (reflected by a significant interaction effect; Fig. 2C,F,I). (4) Finally, if both species co-occur over a considerable portion of their distribution ranges, competition could be another driver of phenotypic divergence (see Supplementary Fig. S3). This ecological character displacement (ECD) has been described for various systems in which congeneric and ecologically similar taxa form secondary contact zones^60^,^61^,^62^.

In summary, we used an integrated analytical framework to tackle several questions related to the coexistence of both species, as well as phenotypic divergence along an environmental gradient in the Río Grijalva. Specifically, we assessed several abiotic and biotic environmental variables at ten sites across a stretch of approximately 100 km in the Río Grijalva, established fish community structures, and assessed morphological and life-history variation in *Gambusia* spp. to answer the following questions: (1) What environmental factors predict the distribution of *G. sexradiata* and *G. yucatana*? (2) Do we find gradient evolution in life histories and morphology in line with *a priori* predictions (life-history variation^21^; body-shape variation^33^,^36^), and, if so, do both species show shared or unique patterns of phenotypic divergence? (3) Which component(s) of the river gradient (including differences in temperature and water depth, predation, etc.) drive divergence in different trait suites?

## Results

### Molecular and phenotypic species identification

#### Phylogenetic analysis

Bayesian phylogenetic analysis of the cyt*b* fragment for two individuals from each population confirmed the presence of both species, *G. sexradiata* and *G. yucatana*, in our dataset. Phylogenetic relationships to representatives of other *Gambusia* species were in line with previously published phylogenies^46^,^47^, even though the rather short cyt*b* fragment yielded only minor support for several divergence events (Fig. 3B). Our analysis confirmed the close relationship between *G. puncticulata* and *G. yucatana*, with the latter often being treated as a subspecies of *G. puncticulata*^63^. Interestingly, the hydrogen sulphide-spring endemic *G. eurystoma* clustered within the sampled specimens of *G. sexradiata.*

### Population genetic analyses

In a second step we amplified nuclear microsatellites and conducted population genetic analyses to verify species identity of the *n* = 239 genotyped individuals. We detected *K* = 2 as the uppermost hierarchical level of population structure according to Evanno *et al*.^64^. Considering those individuals included in the phylogenetic and population genetic analyses we found that the two major genetic clusters in the STRUCTURE analysis correspond to *G. sexradiata* (orange) and *G. yucatana* (green; Fig. 3C). The second highest Δ*K* was found for *K* = 3, followed by *K* = 6 (see Supplementary Fig. S1). The pattern of individual assignment into three and six subpopulations, respectively, revealed population genetic structure within *G. sexradiata*, but not in *G. yucatana*.

Descriptive statistics for site-specific means of standard indicators of genetic variability are provided in Supplementary Table S4. We found significantly higher allelic richness (*A*), expected (*H*_E_) and observed heterozygosity (*H*_O_) in *G. sexradiata* (*A* = 3.8, *H*_E_ = 0.61, *H*_O_ = 0.50) compared to *G. yucatana* (*A* = 3.1, *H*_E_ = 0.49, *H*_O_ = 0.37; Wilcoxon signed-rank tests comparing both species across loci, in all cases: *z* < −2.49, *p* < 0.05; see Supplementary Table S4).

### Test for hybridization

When we tested for a potential hybrid zone between both species (Fig. 2D–F), the software NEWHYBRIDS identified most individuals (98.7%) to be either ‘pure’ (*Q* ≥ 0.95) *G. sexradiata* (169 individuals, 70.7%) or ‘pure’ *G. yucatana* (67 individuals, 28.0%). Only three individuals (1.3%) were of putative mixed ancestry (i.e. *Q* < 0.95). Those individuals, however, could not be unambiguously identified as F_1_- or F_2_-hybrids, or backcrosses with either parental species (*Q* ≤ 0.49). For example, the individual with lowest probability of being a ‘purebred’ (female no. 10 from site 5; *Q*-value for assignment to *G. yucatana* = 0.47; *Q*-value for being an F_2_-hybrid = 0.04; *Q*-value for being a backcross to *G. yucatana* = 0.49; marked by an asterisk in Fig. 3C) was heterozygous at locus Mf13, with two alleles of 167 and 177 bp length, and the 177 bp allele was otherwise exclusive to *G. sexradiata* in our dataset. Thus, hybridization cannot be ruled out entirely; however, it is evidently not frequent.

### Species identification based on external characteristics

**Figure 1:**
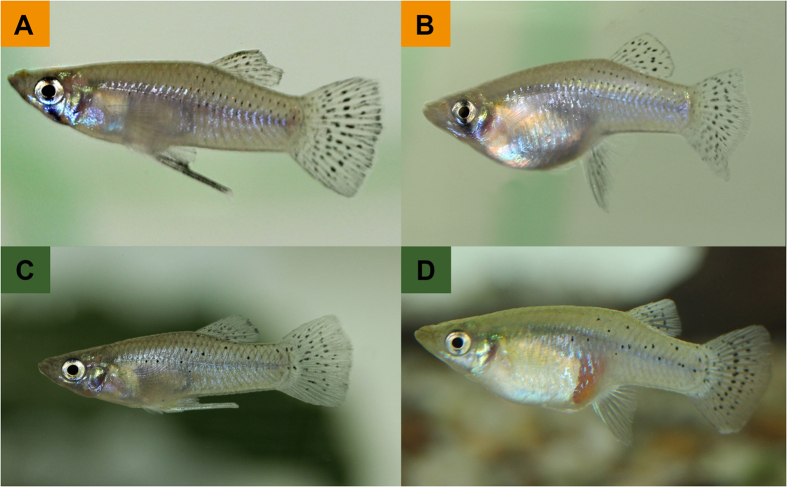
Representative photographs of aquarium-reared *Gambusia* spp. (**A**) male, and (**B**) female *G. sexradiata* (orange) from site 7 and *G. yucatana* (green), (**C**) male and (**D**) female from site 8. Note different pigmentation patterns that allowed us to unambiguously distinguish both species: in *G. sexradiata* lateral black spots are arranged in rows on the dorsal half of the body, while *G. yucatana* displays scattered black spots on the dorsal half of the body.

Application of different criteria described in identification keys^55^ found only pigmentation patterns to accurately distinguish *G. sexradiata* from *G. yucatana*: lateral black spots are arranged in rows on the dorsal half of the body in *G. sexradiata*, while *G. yucatana* has scattered black spots at the dorsal half of the body (Fig. 1). Using this criterion we verified species assignment for all individuals to either the *G. sexradiata* (*n* = 169) or the *G. yucatana* cluster (*n* = 70), i.e., according to the most likely assignment in the STRUCTURE analysis (see above). By comparison, when we used caudal fin spots as a criterion to distinguish species (described as ‘heavily peppered’ in *G. sexradiata*, while caudal fins were described as ‘usually crossed by 1–3 rows of spots’ in *G. yucatana*^55^), only 83.3% of individuals could be correctly assigned. We, therefore, used lateral black spot colour patterns for species delimitation in case of individuals not included in the molecular analyses but included in subsequent analyses.

### Species distributions patterns

**Figure 2:**
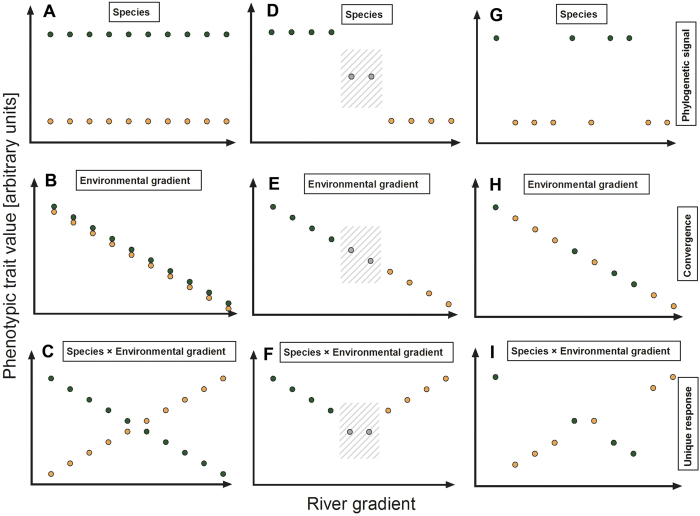
Illustration of hypothetical effects of shared and unique phenotypic trait divergence along a stream gradient in two ecologically competing species (green and orange). (**A**–**C**) Both species occur syntopically along the river gradient. (**A**) Unique responses could arise from different evolutionary histories of both species (i.e., represent a phylogenetic signal) and are thus independent of the river gradient, (**B**) selective forces could result in convergent (shared) patterns of divergence in both species, or (**C**) phenotypic diversification could be due to unique (species-specific) responses to components of the river gradient. (**D**–**F**) Alternatively, the river gradient altogether could determine species distributions, with a potential overlap zone in between, in which hybridization could occur (grey). Again, phenotypic differences could reflect (**D**) a phylogenetic signal, (**E**) shared patterns of gradient evolution, or (**F**) species-specific responses (not illustrated here is the potential outcome of ecological character displacement, where both species diverge in opposing directions in the overlap zone; for illustration see Supplementary Fig. S3). (**G**–**I**) Moreover, certain components of the river gradient could determine small-scale species distribution patterns, leading to a patchy occurrence of both species. Also under this scenario, the same general patterns of gradient evolution can be predicted. Boxes indicate significant effects of the main factor (‘species’), the covariate (‘environmental gradient’), or their interaction in analyses of covariance [(M)ANCOVA] using phenotypic trait values as the dependent variable.

**Figure 3:**
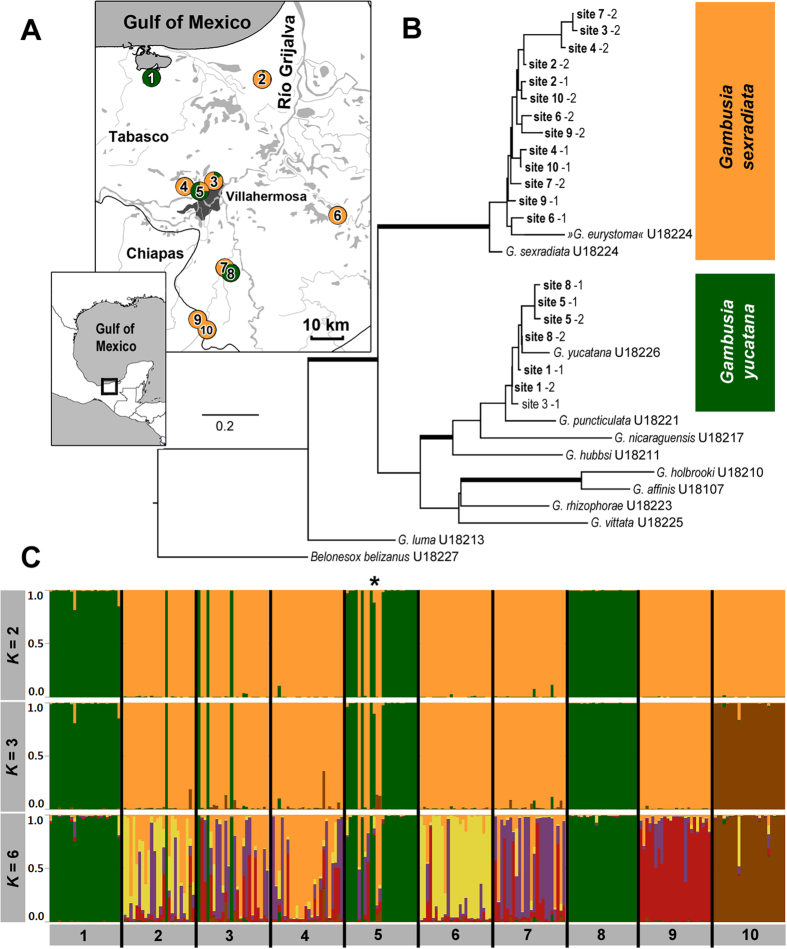
Sample sites and molecular species identification. (**A**) Sampling sites at which *Gambusia sexradiata* (orange) and *G. yucatana* (green) were collected in the Río Grijalva basin. The insert shows the location of our study area in Mexico. At sites 2, 3, and 5 both species were found to occur syntopically, and proportions of occurrence are indicated by different colour coding. The map was generated using DIVA-GIS 7.5^119^. (**B**) Phylogenetic relationships between exemplary individuals from all sampling sites and reference samples from GenBank^47^ inferred using a Bayesian phylogenetic approach based on cyt*b* sequences (maximum clade credibility tree). Branches with posterior probability >0.95 are given in bold. (**C**) Results from STRUCTURE^105^ based on fragment length polymorphisms of 15 nuclear microsatellites. *K* = 2 was the most likely number of genetically distinct clusters according to the method provided by Evanno *et al*.^64^, followed by *K* = 3 and *K* = 6 (see Supplementary Fig. S1). Each individual is represented by a vertical bar, which is partitioned into *K*-coloured segments representing its estimated likelihood of membership (*Q*) to each of the identified clusters. The asterisk marks an individual of putative hybrid origin according to the NEWHYBRIDS analysis (for details see main text).

We tested three different predictions regarding the distribution of both congeners (Fig. 2) and found the majority of sites (seven out of ten) to harbour only one *Gambusia* species, while both species occurred syntopically at sites 2, 3, and 5 (Fig. 3A). There was no obvious pattern of zonation (Fig. 3A), whereby *G. sexradiata* might be restricted to upstream, and *G. yucatana* to downstream portions along the stream gradient (Fig. 2D–F).

#### Canonical correspondence analysis: community compositions

**Figure 4:**
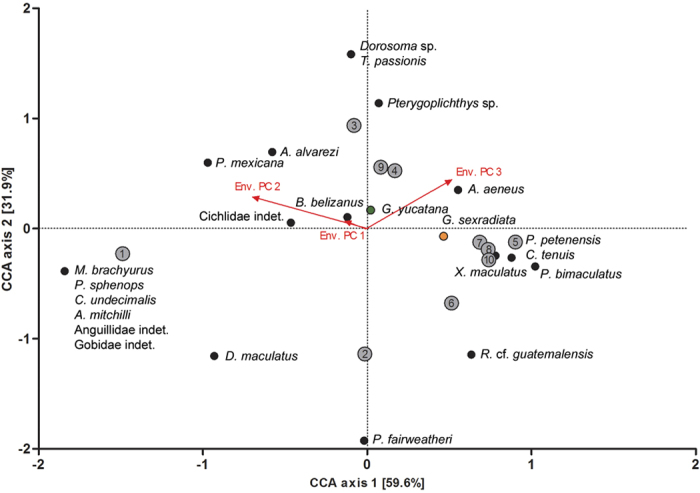
Fish community structure across sample sites. Results from canonical correspondence analysis (CCA) showing the effects of environmental variables (‘environmental PCs’, see main text) on fish community structures using occurrence data (present/absent) of different teleosts as the dependent data matrix (see Supplementary Table S2). Species are marked by black circles; grey circles indicate the position of sample sites. Length and direction of arrows indicate the relative importance and direction of the environmental variables.

We asked whether environmental factors explain the distribution of the two congeners. Our first canonical correspondence analysis (CCA) using presence/absence data of all teleost species per site explored the effects of environmental factors on local fish community compositions (Fig. 4). A permutation test (*p* = 0.04) suggested that a significant portion of the variance in community compositions could be ascribed to variation along the three environmental PCs. The first two axes of the CCA ordination map explained 91.5% of the cumulative (constrained) variance (axis 1, eigenvalue = 0.65, 59.6% variance explained; axis 2, eigenvalue = 0.35, 31.9% variance explained; Fig. 4). The first axis ordered sample sites along a gradient from large, deep and more coastal water bodies with high predation pressure to shallow inland habitats with low predation pressure (Fig. 4). The second axis ordered sample sites from deep inland waters to shallow, more coastal water bodies. Fish communities changed from species-rich coastal assemblages including marine species, to inland communities with a lower α-diversity, often characterized by the presence of platyfish (*Xiphophorus maculatus*) and dogtooth rivulus (*Cynodonichthys tenuis*; Fig. 4; see Supplementary Table S2).

#### Canonical correspondence analysis: distribution of *Gambusia* spp

Both *Gambusia* species clustered closely together in our first CCA, and we found only a minor shift of *G. sexradiata* towards a more negative position along environmental PC 2, suggesting a somewhat higher likelihood of occurrence at sites with lower water depth, reduced predation pressure, and lower salinity and conductivity (Fig. 4). Furthermore, compared to *G. yucatana, G. sexradiata* adopted a position along more positive values of environmental PC 3, suggesting that the latter species occurs at sites with higher maximum temperatures and a higher annual temperature range.

Caution is required when interpreting the results from our first CCA in light of the outcome of our second CCA, which analysed the influence of environmental factors on the local abundance of both *Gambusia* species. In this analysis, we did not find any evidence that the environmental factors would explain the occurrence of both congeners. The first and only axis of the CCA (eigenvalue = 0.16) explained 19.6% of the cumulative (constrained) variance, and we found no significant effect of the three environmental PCs on species distribution patterns (permutation test, *p* = 0.71).

### Phenotypic divergence

Based on the observed distribution patterns (see above), we proceeded with analyses of phenotypic trait divergence according to the predictions outlined in Fig. 2A–I. Tests of a signature of ecological character displacement (ECD; see Supplementary Fig. S3) were not possible because only few sites harboured both species. The results from MANCOVAs testing conflicting predictions of gradient evolution (Fig. 2A–I) found support for (*a*) major species differences (significant ‘species’ effects on male and female life histories and body length in both sexes, but notably only on female, but not male body shape variation; Table 1), (*b*) shared responses to at least some components of the river gradient (main effects of environmental PCs 1 through 3, which were found in all analyses), and (*c*) unique (species-specific) responses, as indicated by significant interaction terms of ‘species’ and environmental PCs 1 through 3 (Table 1). In the following we will discuss the results from trait-specific univariate ANCOVAs (see Supplementary Tables S5–S8) that were conducted *post hoc* for all significant effects in our main MANCOVA models (Table 1).

#### Species differences in morphology and life histories

**Figure 5:**
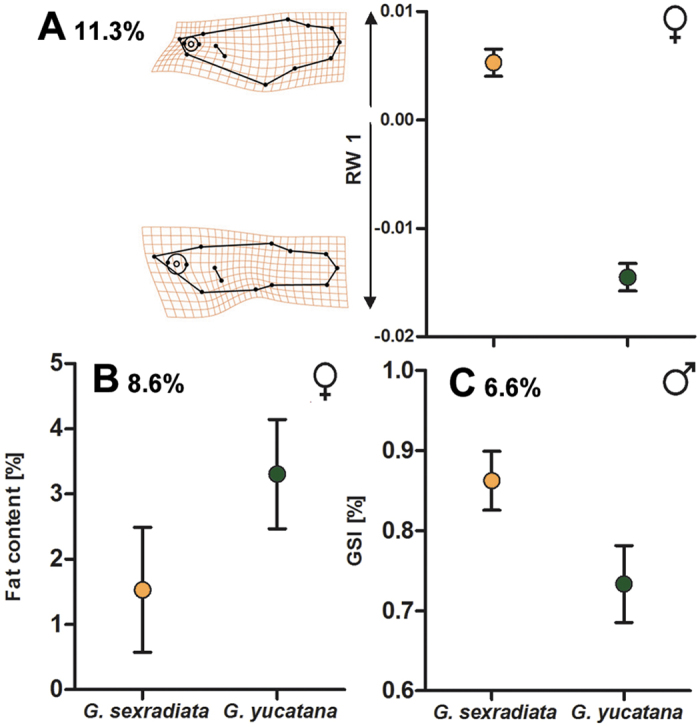
Graphic illustration of three traits for which a main effect of species identity was uncovered. This suggests that differences arose from different evolutionary histories of both taxa (i.e., represent a phylogenetic signal) and are independent of the environmental gradient (Table 1): (**A**) *G. sexradiata* females had a deeper body and smaller head size compared to *G. yucatana*, as indicated by a significant effect on relative warp (RW) 1, (**B**) female fat content was lower in *G. sexradiata* than in *G. yucatana*, and (**C**) male GSI was higher in *G. sexradiata* than in *G. yucatana*. Relative variance explained (in percent) is shown for each factor.

In the MANCOVAs, we found only a small proportion of phenotypic variance to be explained by species identity [relative variance explained (*V*_rel_) for female body shape = 16.6%, male life histories = 8.9%, female life histories = 13.6%; Table 1]. In the subsequent trait-wise ANOVAs, species differences became apparent in three cases: *G. sexradiata* females had a deeper body and smaller head size compared to *G. yucatana* females (*V*_rel_ = 11.3%; Fig. 4A; see Supplementary Table S6–S8). Furthermore, female fat content was lower in *G. sexradiata* than in *G. yucatana* (*V*_rel_ = 8.6%; Fig. 5B), and male GSI was higher in *G. sexradiata* than in *G. yucatana* (*V*_rel_ = 6.6%; Fig. 5C).

#### Shared patterns of divergence

We found shared patterns of body-shape and life-history divergence only in female (but not male) *Gambusia* spp. (see Supplementary Tables S6 and S8). The MANCOVAs revealed that the shared component of female body-shape and life-history divergence could be explained by environmental PC 2 (*V*_rel_ = 50.5% and *V*_rel_ = 36.5%, respectively) and environmental PC 3 (*V*_rel_ = 43.0% and *V*_rel_ = 13.0%, respectively). Furthermore, the ANCOVA examining body length in both sexes showed a strong effect of environmental PC 2 on shared patterns of body length divergence (*V*_rel_ = 34.4%).

##### Environmental PC 2

**Figure 6:**
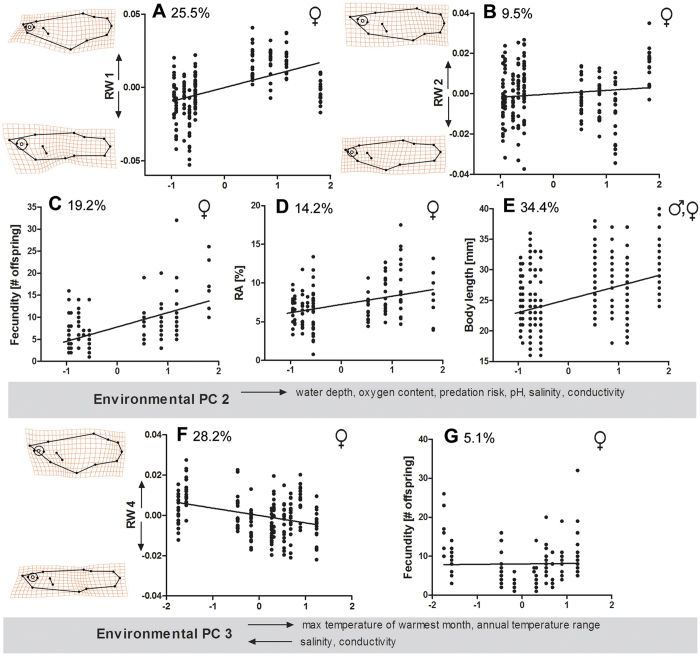
Graphic illustration of shared patterns of phenotypic divergence, suggesting that selective forces along the stream gradient result in convergent patterns of differentiation in both species. With increasing values of environmental PC 2, (**A**) female *Gambusia* spp. showed deeper bodies and smaller heads [as indicated by a significant effect on relative warp (RW) 1], (**B**) females slightly decreased caudal peduncle length and increased head size (RW 2), (**C**) females increased fecundity as well as (**D**) RA, and (**E**) both sexes increased body length. Increasing values of environmental PC 3 resulted in (**F**) slender bodies in females (RW 4) and (**G**) a slightly higher female fecundity. Relative variance [%] is given for each variable (see also Supplementary Tables S5–S8).

Environmental PC 2 describes the gradient from shallow water bodies, with low oxygen levels and low predation risk, lower pH, salinity and conductivity towards deeper water bodies, with high oxygen content and increased predation risk (Table 2). We found a strong effect of environmental PC 2 on components of female body morphology, namely relative warp (RW) 1 (*V*_rel_ = 25.5%). This effect can be interpreted as females of both species evolving a deeper body and relatively smaller heads in bigger, deeper water bodies and under increased predation risk, while females were more slender-bodied in shallow water bodies with low predation risk (Fig. 6A). Moreover, we found a slight increase in RW 2 along environmental PC 2 (*V*_rel_ = 9.5%) across species, which can be interpreted as caudal peduncle lengths becoming smaller with higher values of environmental PC 2 (Fig. 6B). Regarding female life-histories we found a strong shared response of increasing fecundity along environmental PC 2 (*V*_rel_ = 19.2%), suggesting that females produced more offspring per clutch when exposed to higher predation risk in deeper water bodies (Fig. 6C). Furthermore, reproductive allocation (RA) increased significantly across species along environmental PC 2 (*V*_rel_ = 14.2%; Fig. 6D). Our analysis of body length uncovered a strong increase of body length along environmental PC 2 (*V*_rel_ = 34.4%; Fig. 6E).

##### Environmental PC 3

Environmental PC 3 describes the gradient from coastal waters, with high salinity and conductivity, towards more inland waters with higher maximum temperatures and a high annual temperature range. Along this gradient, females of both species showed strong divergence in RW 4 (*V*_rel_ = 28.2%), suggesting deeper bodies with increasing salinity and conductivity (Fig. 6F). Additionally, a weak shared response of increasing fecundity along environmental PC 3 was detected (*V*_rel_ = 5.1%; Fig. 6G).

#### Unique patterns of body-shape and life-history divergence

In both sexes, we found several cases of unique (species-specific) responses to environmental variables in body-shape and life-history divergence (Table 1; see Supplementary Tables S5–S8). The MANCOVAs revealed that male and female shape variation was influenced by the interaction terms ‘species × environmental PC 1’ (*V*_rel_ = 22.9% and *V*_rel_ = 21.1%, respectively) and ‘species × environmental PC 3’ (*V*_rel_ = 28.8% and *V*_rel_ = 17.4%, respectively). For male life-history divergence, we found significant interaction effects of ‘species × environmental PC 1’ (*V*_rel_ = 20.0%), ‘species × environmental PC 2’ (*V*_rel_ = 9.5%) and ‘species × environmental PC 3’ (*V*_rel_ = 6.1%), while for female life-history divergence, only the interaction terms ‘species × environmental PC 1’ (*V*_rel_ = 15.8%) and ‘species × environmental PC 2’ (*V*_rel_ = 14.7%) were significant.

##### Environmental PC 1

**Figure 7:**
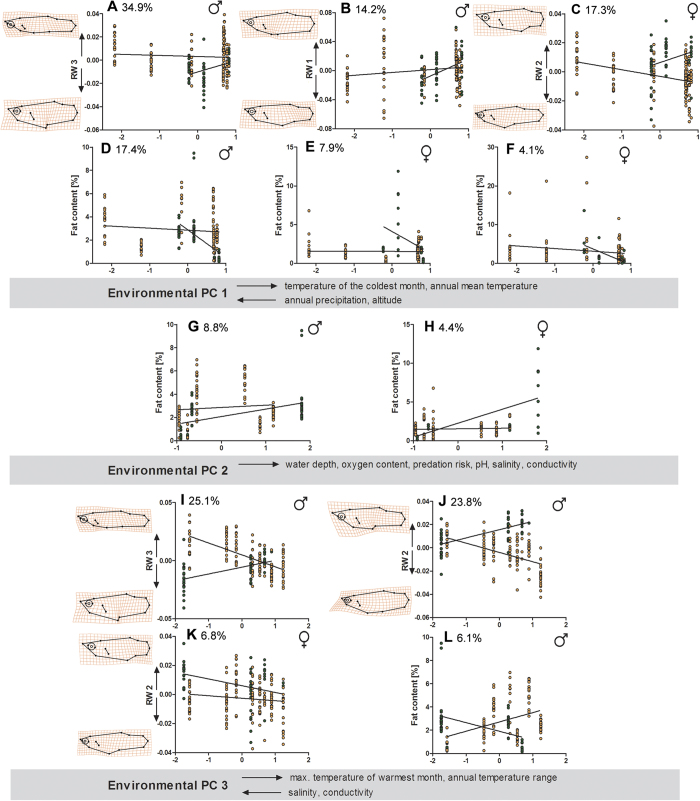
Graphic illustration of unique (species-specific) patterns of phenotypic divergence in *Gambusia sexradiata* (orange) and *G. yucatana* (green). Increasing values along ‘environmental PC 1’ (Table 2) resulted in (**A**) slightly decreased body depth (RW 3) of male *G. sexradiata*, while *G. yucatana* increased body depth, (**B**) slight (*G. sexradiata*) versus strong (*G. yucatana*) shifts towards a more anterior position of the gonopodium (RW 1), (**C**) decreased caudal peduncle length and increased head size in female *G. sexradiata*, but opposing patterns in *G. yucatana* (RW 2), (**D**) slightly (*G. sexradiata*) or strongly (*G. yucatana*) decreased male fat content, (**E**) virtually no change in female fat content (*G. sexradiata*) versus strongly decreased fat content (*G. yucatana*), and (**F**) slightly (*G. sexradiata*) versus strongly (*G. yucatana*) decreased embryo fat content. With increasing values along ‘environmental PC 2’, (**G**) male fat content slightly increased in *G. sexradiata* while the increase was more pronounced in *G. yucatana*, and (**H**) female fat content showed an increase only in *G. yucatana*. Increasing values along ‘environmental PC 3’ resulted in: (**I**) *G. sexradiata* males being deeper-bodied, while *G. yucatana* males were more slender-bodied (RW 3), (**J**) male *G. sexradiata* decreasing head size, while *G. yucatana* increased head size (RW 2), (**K**) *G. sexradiata* females slightly and *G. yucatana* females strongly decreasing caudal peduncle length and increasing head size (RW 2), and (**L**) male fat content increasing in *G. sexradiata*, whereas it decreased in *G. yucatana.* Relative variance [%] is given for each interaction term (see also Supplementary Tables S5–S8).

Environmental PC 1 describes the gradient from colder waters at high altitudes with high annual precipitation towards lowland water bodies with higher temperatures. We found a strong species-specific pattern of divergence of male RW 3 along environmental PC 1 (*V*_rel_ = 34.9%). This effect can be interpreted as male *G. sexradiata* being more slender-bodied at higher altitudes, while *G. yucatana* males showed the opposite pattern, with more slender-bodied males found at lowland sites (Fig. 7A). Male RW 1 increased slightly with environmental PC 1 in *G. sexradiata* but increased strongly in *G. yucatana* (*V*_rel_ = 14.2%). The position of the gonopodium changed only little in *G. sexradiata*, while it adopted a more anterior position on the body at sites with higher mean annual temperature in *G. yucatana* (Fig. 7B). Female RW 2 decreased with environmental PC 1 in *G. sexradiata* but increased in *G. yucatana* (*V*_rel_ = 17.3%), suggesting that *G. sexradiata* females decreased caudal peduncle length and increased head size along environmental PC 1, while *G. yucatana* showed the opposite pattern (Fig. 7C). Male fat content decreased slightly with environmental PC 1 in *G. sexradiata* but decreased strongly in *G. yucatana* (*V*_rel_ = 17.4%; Fig. 7D). Female fat content did not change along environmental PC 1 in *G. sexradiata*, but strongly decreased in *G. yucatana* (*V*_rel_ = 7.9%; Fig. 7E). Embryo fat content decreased slightly along environmental PC 1 in *G. sexradiata*, but strongly in *G. yucatana* (*V*_rel_ = 4.1%; Fig. 7F). However, *G. yucatana* was largely restricted to sites in the upper part of the distribution range along environmental PC 1, and so the significant interaction effects must be interpreted with caution in all cases.

##### Environmental PC 2

We found only few cases of unique responses along environmental PC 2, which received positive axis loadings, among other factors, from water depth and predation risk (Table 2). Male fat content increased slightly with environmental PC 2 in *G. sexradiata* while the increase was more pronounced in *G. yucatana* (*V*_rel_ = 8.8%; Fig. 7G). A similar pattern was found for female fat content, for which *G. sexradiata* showed no response along environmental PC 2, while fat content increased in *G. yucatana* females (*V*_rel_ = 4.4%; Fig. 7H).

##### Environmental PC 3

We found strong patterns of species-specific divergence along environmental PC 3, which describes the gradient from coastal waters, with high salinity and conductivity, towards more inland waters with higher maximum temperatures and a high annual temperature range. Along this gradient we found unique patterns of variation in male body shape (*V*_rel_ = 25.1%), with *G. sexradiata* being more slender-bodied at coastal sites and deeper-bodied in inland habitats, while *G. yucatana* displayed the opposite pattern, with deeper-bodied specimens being found at coastal sites and slender-bodied inland populations (Fig. 7I). In male *G. sexradiata* we found a decrease of RW 2 along environmental PC 3, while RW 2 increased in *G. yucatana* (*V*_rel_ = 23.8%). This effect can be interpreted as male *G. sexradiata* decreasing head size from coastal to inland waters, while *G. yucatana* showed the opposite pattern of divergence (Fig. 7J). Females showed species-specific shape divergence in RW 2 (*V*_rel_ = 6.8%), reflecting that caudal peduncle lengths became smaller with increasing environmental PC 3, with a less pronounced decrease in *G. sexradiata* compared to a much stronger decrease in *G. yucatana* (Fig. 7K). Finally, male fat content increased in *G. sexradiata* along environmental PC 3 while it decreased in *G. yucatana* (*V*_rel_ = 6.1%; Fig. 7L).

#### Maternal provisioning (matrotrophy index)

To evaluate the mode of maternal provisioning, we calculated matrotrophy indices (MI)^65^,^66^. Maternal provisioning of embryos may be entirely through yolk deposited in the unfertilized egg (lecithotrophy, MI between 0.6 and 0.7) or may include post-fertilization nutrient transfer to varying degrees (matrotrophy, MI > 0.7). The GLM detected no effect of species identity (*F*_1,3_ = 0.32, *P* = 0.61, *V*_rel_ = 13.5%), and estimated MI-values (across populations) were similar between *G. sexradiata* (MI = 0.941) and *G. yucatana* (MI = 0.889; see Supplementary Fig. S5). We detected some degree of variation among sites, whereby some populations showed little to no maternal provisioning (0.5 > MI < 0.7), while others showed moderate amounts of maternal provisioning (0.7 > MI < 1.2; Supplementary Fig. S6). When statistically comparing whether or not there was maternal provisioning after fertilization^65^,^66^, MI was significantly greater than 0.7 in two populations (Population 2: *z*_16_ = 2.16, *p* = 0.031; Population 3: *z*_15_ = 2.09, *p* = 0.036), and there was a non-significant trend in population 7 (*z*_10_ = 1.77, *p* = 0.077). However, our GLM found no predictable pattern of diversification along the three environmental PCs (environmental PC 1: *F*_1,3_ = 0.45, *p* = 0.55, *V*_rel_ = 18.3%; environmental PC 2: *F*_1,3_ = 0.14, *p* = 0.74, *V*_rel_ = 6.1%; environmental PC 3: *F*_1,3_ = 0.16, *p* = 0.71, *V*_rel_ = 7.2%).

## Discussion

Phylogenetic relationships uncovered in our present study supported our assessment of species identity inferred on the basis of colour patterns and confirmed the proposed close relationship between *Gambusia yucatana* and *Gambusia puncticulata*^47^. *Gambusia eurystoma*—a species that is endemic to a hydrogen sulphide-rich spring complex^48^,^49^,^50^—clustered within *G. sexradiata*, which mirrors the results of a previous study^67^. This further highlights the need for additional investigations to assess gene flow and the degree of population genetic differentiation in this system^45^,^46^,^47^, which was beyond the scope of this study. Specifically, it appears as if ‘*G. eurystoma*’ could represent a case of incipient ecological speciation as a locally-adapted ecotype of the widespread *G. sexradiata*. Similar patterns were described for sulphide-adapted populations in the *Poecilia mexicana*-species complex, with phylogenetically old lineages (*P. sulphuraria, P. thermalis*) occurring in some sulphide springs, while others are inhabited by phylogenetically young sulphide-adapted ecotypes of *P. mexicana*^68^.

Compared to *G. sexradiata*, we found a lower genetic variability and no population genetic structure in *G. yucatana*, which could be an indicator of a more recent invasion of the Río Grijalva system by the latter species*. Gambusia yucatana* is a member of the *Gambusia puncticulata-*species group, the members of which are distributed across the Greater Antilles^45^,^46^,^47^. Given its tolerance to sea water^57^, it is well conceivable that the species invaded Mexican watersheds through marine long-distance dispersal, e.g., during hurricanes^69^. Such a close Caribbean–Central American relationship of freshwater biota also becomes evident for other groups of organisms, e.g., freshwater crabs^70^. *Gambusia sexradiata* is part of the *Gambusia nobilis*-species group, all extant members of which occur on the North American mainland. *Gambusia sexradiata* is the only member with a wide distribution range, and it is the only member of the clade that is not entirely restricted to freshwater environments^45^,^46^. Altogether, the evolutionary histories of both species suggest a competitive advantage of *G. yucatana* at coastal sites, while *G. sexradiata* should be better adapted to inland conditions; which would also be consistent with previous reports on their distributions^45^,^55^,^57^.

Contrary to prediction, we found a patchy occurrence of *G. sexradiata* and *G. yucatana* along the investigated stream gradient, with syntopic occurrences at only few sites. Both species occupied a wide range of environmental conditions regarding pH, dissolved oxygen and conductivity, and we did not identify any ecological factor that might limit the distributions of both congeners. *Gambusia sexradiata* and *G. yucatana* are adapted to slow-flowing or stagnant conditions^45^,^56^ and are probably more affected by floods compared to species like *Poecilia mexicana* and *Astyanax aeneus*, both of which are adapted to higher stream velocities^16^,^56^. Contrary to other river systems of Mexico, damming projects were not as extensive in the Grijalva basin, and so the stream still experiences regular catastrophic flooding^44^. This may partly determine the seemingly stochastic distribution of both species along the river gradient, since massive dislocations during floods leave few individuals at a given site and subsequently allow either species to build up local populations again.

With the exception of three doubtful cases, we found no indication for extensive hybridization between species, suggesting reproductive isolation by means of behavioural (pre-mating) isolation, mechanical mating incompatibilities (for different gonopodial structures see ref. 53), or gametic incompatibilities (for review see ref. 71). For example, a unidirectional post-reproductive barrier by means of different chromosomal sex-determination has evolved between the closely related *G. affinis* (heteromorphic WZ–ZZ system) and *Gambusia holbrooki* (XX–XY)^72^,^73^. Future studies will need to shed light on the mechanisms of reproductive isolation at play in the system studied here.

We found shared phenotypic responses in components of body-shape and life-history divergence that were driven mainly by variation in predation regimes (included in ‘environmental PC 2’), and which followed patterns of phenotypic diversification along ecological gradients of predation risk described for other poeciliids (shape divergence^33^,^35^,^74^, life history divergence^21^,^23^,^66^). Interestingly, we found shared patterns of body-shape and life-history divergence only in female (but not male) *Gambusia* spp. It appears as if female body shape and life-history traits are a common target of selection, while males are responding in a species-specific way (see below). As described for other mosquitofishes^56^,^75^, male *Gambusia* spp. (SL, mean ± SE: 23.26 ± 0.30 mm) in our study were substantially smaller than females (28.47 ± 0.43 mm), possibly translating into a much stronger predation risk for females, as avian, piscine and invertebrate predators may favour large-bodied poeciliids^21^,^76^. Furthermore, males of most *Gambusia* species do not show flamboyant secondary sexual colour ornaments like male guppies (*P. reticulata*), which therefore suffer higher mortality from predation compared to female guppies^19^ (but see ref. 77 for sexual ornamentation in *G. hubbsi*).

We found females of both species to evolve relatively smaller heads (RW 1) and a deeper caudal peduncle (which was, however, not longer, as reported for other poeciliids^33^,^36^,^74^) in bigger, deeper water bodies and under increased predation risk, while females were more slender-bodied in shallow water bodies with low predation risk. Similar observations were made by Langerhans and Makowicz^78^, who reported deeper bodies, but no increased caudal peduncle length in *G. caymanensis* females in response to increased predation risk. The deeper caudal region and the relatively small head are trait values associated with improved fast-start performance, while the short, but deep caudal peduncle is associated with increased steady-swimming performance and likely increases manoeuvrability in bigger water bodies^74^. Likewise, increased fecundity and RA under high predation risk was reported for several poeciliids, including *P. reticulata*^21^,^23^, *Brachyrhaphis rhabdophora*^79^, and *G. hubbsi*^66^. This is in congruence with life-history theory, which predicts that when adult mortality rates are high relative to juvenile mortality rates, females increase reproductive effort, investing in as many offspring per clutch as possible to maximize fitness, even if this investment comes at the cost of producing smaller offspring^25^,^80^.

Previous studies mostly considered divergence of single traits (i.e., either shape or life history divergence) along stream gradients. Our analytical framework enabled us to consider the interplay of multivariate variation in several character suites. For example, body-shape divergence between predator regimes in females might reflect a trade-off between selection on locomotion and selection on reproduction^81^,^82^,^83^. Selection might act primarily on reproductive traits (fecundity, RA) that indirectly affect body shape. Hence, a possible explanation for the unexpectedly short, but deep caudal region under increased predation risk could be that selection favours large abdominal regions that provide space for an increased number of embryos. Similar observation of how life histories might influence body shape were made in other studies on livebearing fishes. For example, Wesner *et al*.^83^ demonstrated that body shape differences between high- and low-predation populations of *B. rhabdophora* largely disappear in gravid females, while two other recent studies showed that high RA negatively affects swimming performance and predator escape responses in guppies^81^,^84^, and thus results in a change in habitat use to compensate for this constraint on swimming performance^84^. Similar observations were made in *Drosophila melanogaster*, in which selection on fecundity also led to rapid morphological change^85^. In our system, such changes may also come at a cost as a shorter caudal region precludes higher burst-swimming speed—normally favoured when prey species coexist with piscine predators^33^,^74^. Future investigations should focus on the correlation of phenotypic traits by selectively altering single environmental parameters (e.g., predation risk, water depth, velocity) to uncover potential patterns of correlated phenotypic evolution. This would also rule out an alternative explanation of our findings, that selection favours females with good steady-swimming abilities in bigger water bodies. These traits might become manifest also under increased predation pressure due to the greater energetic requirements of increased RA and fecundity (i.e. more time spent foraging). This would explain short caudal regions, a trait associated with increased steady-swimming performance^74^.

Furthermore, we found increased body length in both sexes in deeper water bodies and under higher predation risk. Lowland waters are characterized by higher accumulation of nutrients, higher photosynthetic primary production, more stable conditions and higher conspecific densities^17^,^20^,^21^,^22^,^23^,^24^. Bisazza and Marin^86^ found bigger *G. holbrooki* males to have higher reproductive success under high population densities, since they monopolize access to females, while small males had an advantage when interactions with other males were rare. Therefore, high intraspecific competition should favour increased body size and lateral projection areas, especially in deep water bodies with more stable population densities^86^,^87^. This explanation is also congruent with the increased body depth in females of both species along environmental PC 3, suggesting deeper bodies with increasing salinity and conductivity (Fig. 6F). Deep-bodied populations were previously described for *G. yucatana* from localities close to the sea^54^, and we could confirm those patterns not only for *G. yucatana* (Figs 6F and 7I) but also for *G. sexradiata* (Fig. 6F). However, in *G. sexradiata* only females were more deep-bodied at coastal sites, while males showed the opposite pattern with deeper bodies at inland sites (Fig. 7I). It is tempting to argue that this reflects more stable population densities (and higher competition) in *G. sexradiata* at inland sites, a scenario supported by other findings from our present study (see below). Instead, body depth of females does not seem to be density-dependent and rather reflects higher manoeuvrability (deep body, short caudal regions; Fig. 7F) in bigger water bodies^74^.

We found unique (species-specific) phenotypic responses in components of body-shape and life-history divergence that were driven mainly by abiotic conditions (climatic conditions, salinity, conductivity), and were particularly strong in males. Unique patterns of diversification were most pronounced along environmental PC 3, which describes the gradient from coastal waters, with high salinity and conductivity, towards more inland waters with higher maximum temperatures and a high annual temperature range. This is further evidence that patterns of local adaptation in poeciliid fishes are often sex-specific^88^,^89^,^90^. While we do not have a compelling explanation for the patterns uncovered here, we propose that they might be based on differences in intra- and intersexual selection between these two species, which is known to act strongly on males^91^,^92^ and is often population-specific^93^,^94^,^95^. This combination might lead to slightly idiosyncratic patterns in males, which would be absent in females, which are more strongly affected by natural selection.

Differences in potentially fitness-related traits like fat content as a function of environmental PCs 1, 2, and 3 were slightly contradictory with respect to some environmental variables, as they would suggest almost opposite patterns of fat content along the gradient from high-altitude inland populations to low-altitude and more coastal populations. However, when investigating patterns of fat content specifically in response to ‘distance to the sea’ (see Supplementary Fig. S4), it became apparent that in males, patterns were opposite for both species, with fat content in *G. sexradiata* increasing with increasing distance to the sea, while fat content decreased in *G. yucatana*. For females, patterns of fat content were similar in both species, and fat content decreased with increasing distance from the sea. However, this pattern was again much stronger in *G. yucatana* than in *G. sexradiata* (Supplementary Fig. S4). If such patterns were persistent and not just a temporary pattern caught in our sampling scheme, then this would be congruent with previous reports of their respective distributions^45^,^55^,^57^, which suggested that *G. yucatana* might be better adapted to coastal regions relative to *G. sexradiata*. Future studies will have to investigate this further.

Deeper bodies (Fig. 7I) and bigger bodies (compared to head size, Fig. 7J) correlated with increased fat content (Fig. 7L) and might be a by-product of higher fat content, and thus indicative of a better body condition^96^. Furthermore, more stable population densities of *G. sexradiata* in inland waters are likely to cause higher mate competition among males and favour larger bodies with increased lateral projection area (i.e. body depth; RW 2, RW 3), which is a target of female mating preferences^86^,^87^ (see above).

Finally, caution is required when interpreting gradual phenotypic divergence in multiple character suites as a signal of evolutionary divergence, as the observed phenotypic divergence could reflect both evolved differences and adaptive phenotypic plasticity. For example, Hendry *et al*.^34^ found pronounced differences in body shape between wild-caught *P. reticulata* from two consecutive years of sampling at the same locality, suggesting that plasticity may have contributed to the observed variation. On the other hand, Riesch *et al*.^89^ found strong site-specific life-history divergence in cave-dwelling *P. mexicana* compared to surface-dwelling populations in traits such as fat content, standard length and gonadosomatic index, whereby divergence between these traits was retained after several generations under common garden conditions, suggesting a strong heritable component to the observed divergence. We found pronounced phenotypic differentiation within both species (Figs 6 and 7), while our population genetic data revealed no genetic structure within *G. yucatana* (Fig. 3C; Supplementary Table S3). By contrast, we found population genetic structure within *G. sexradiata*, which did, however, not correlate with phenotypic trait divergence (Supplementary Table S11; Supplementary Fig. S7). This illustrates that phenotypic divergence occurred independent of neutral genetic population differentiation in the latter species, due to phenotypic plasticity and/or site-specific selection on coding loci underlying phenotypic differences.

In conclusion, we formulated conflicting hypotheses for species distribution patterns and potential patterns of phenotypic trait divergence (Fig. 2). We found patchy distribution patterns of both species on a small spatial scale, with one of the two species usually being dominant at a given site. Most of the investigated traits showed gradual variation depending on several environmental factors, whereby females of both species mostly exhibited shared patterns while males exhibited unique patterns of trait divergence in response to the same components of a river gradient (following the predictions illustrated in Fig. 2H and I). Our findings therefore demonstrate the complexity of phenotypic responses even in relatively closely related—and supposedly ecologically very similar—species when exposed to the same environmental variation along a river-system gradient. Our study also illustrates how the interplay between natural and sexual selection seems to affect both sexes in different, sometimes even opposing, ways (both on the level of populations and along the river gradient). Finally, our study was conducted in a river drainage that has, until now, not been affected much by damming projects^44^, which have the potential to affect the evolutionary trajectories of populations^29^,^31^. Our current study demonstrates complex patterns of microevolutionary phenotypic diversification that are threatened by the widespread practice to produce hydroelectric power from stream impoundments^97^.

## Material and Methods

For the collection of the data at hand, the authors have adhered to the Guidelines for the Use of Animals in Research. The current study does not include experiments involving living animals. All collections of specimens from natural populations in Mexico were approved by the Mexican Federal Agency (CONAPESCA: PRMN/DGOPA-003/2014 and PRMN/DGOPA-009/2015).

### Study sites

We collected *Gambusia* spp. (Fig. 1) from 10 sites in the Grijalva Basin in Tabasco, Estados Unidos de México, ranging from coastal lagoons to stagnant waters in the foothills of the Sierra Madre de Chiapas (Table 3; Fig. 3A). We caught fish with dip nets (35 × 35 cm, mesh size 3 mm) during the dry season in April 2014 and 2015, when several stagnant water bodies were separated from the various affluent streams draining the Río Grijalva. All captured specimens were sacrificed with an overdose of clove oil and preserved in 96% ethanol until they were processed in the laboratory for molecular, morphological, or life-history analyses.

Environmental characteristics in the Grijalva Basin change markedly along the longitudinal gradient from the mountainous Sierra Madre de Chiapas to the coastal plains of Tabasco. The upper Grijalva has two large hydroelectric dams that dampen the stream flow regime and remove large amounts of sediment from the system^44^. *Gambusia* spp. typically do not occur in headwaters^43^,^56^, and so our study sites were located below the dams at the lower reaches of the Grijalva basin, where the Grijalva interconnects with the Usumacinta Basin through multiple bifurcating distributaries that form an extensive wetland system. The lower Río Grijalva is strongly affected by human activities, and the petroleum industry has probably created the most substantial change^44^.

### Environmental and climate data

We examined a range of environmental variables that show gradual variation along the examined stretch of the Río Grijalva. At each site, we measured salinity using a Hanna HI 96822 refractometer, and dissolved oxygen [mg L^−1^] and pH using a HACH^®^, HQ40d multimeter (see Supplementary Table S1). We estimated average water depth at our study sites as belonging to one of four categories (<1 m, 1–3 m, 3–5 m, >5 m). We were not able to quantify predation risk but made an attempt to provide an overview of co-occurring (predatory and non-predatory) fishes. First, we conducted predator surveys by slowly walking the survey areas along the shoreline and in shallow parts of the water for at least 30 min and noted all predatory species we sighted (see Supplementary Table S2). Afterwards, four persons collected fishes for at least 30 min per site using a seine (3 m long, 3 mm mesh with). We expressed predation risk as one of three categories (*low*: *Gambusia* spp. co-occurred with the killifish *Cynodonichthys tenuis* or other small, omnivorous poeciliids that do not prey on adult *Gambusia*; *medium*: *Gambusia* spp. co-occurred with cichlid predators or the poeciliid *Belonesox belizanus* that regularly preys on *Gambusia* spp.; *high*: the presence of large-bodied predators of marine origin, like *Centropomus undecimalis*, in combination with large-bodied piscivorous cichlids suggested high predation pressure on all size classes of *Gambusia* spp.).

We downloaded climatic data (averaged from 1950–2000) for each study site from the Worldclim database^98^ at a 2.5 arc-minutes resolution. We included five climatic variables: (*i*) mean annual temperature, (*ii*) maximum temperature of the warmest month, (*iii*) minimum temperature of the coldest month, (*iv*) annual temperature range, and (*v*) annual precipitation. Altitude was extracted from Google Earth (http://earth.google.com/).

We condensed environmental variables through a factor reduction (principal components analysis, PCA) with varimax rotation. The three resulting principle components (PCs; henceforth called ‘environmental PCs’) with an eigenvalue >1.0, explaining 89.1% of environmental variation, were used to characterize the stream gradient along the examined stretch of the Río Grijalva, and were used as covariates in the statistical analyses (Table 2).

### Molecular species identification

For species identification we initially used the keys provided by Greenfield *et al*.^55^. However, *ad hoc* determination of both *Gambusia* species may be difficult, since morphological characteristics, like numbers of fin rays or pigmentation patterns are variable across populations^55^. To verify our assessment of species identity, we therefore used the following approach: First, we generated phylogenetic information for a subset of individuals based on sequence variation of mitochondrial DNA. Second, we amplified nuclear microsatellites for a larger number of individuals (including those for which phylogenetic information was available) and conducted population genetic analyses to verify species identity and to test for potential hybridization. After genotyping, we found pigmentation patterns to be the most accurate criterion to distinguish *G. sexradiata* from *G. yucatana*: lateral black spots are arranged in rows on the dorsal half of the body in *G. sexradiata*, while *G. yucatana* has scattered black spots at the dorsal half of the body (Fig. 1). Individuals classified using this criterion obtained the highest probability of correct assignment in our population genetic analyses (see Results). Therefore, we used this criterion to identify species for all further analyses. Note, however, that this trait appears not to distinguish both species over their entire distribution range^55^.

#### Phylogenetic analysis

We sequenced a 387 bp segment of the mitochondrial cytochrome *b* gene for two specimens from each population (*n* = 20). We extracted DNA from fin-clips using the Nucleo Spin Tissue kit (Macherey-Nagel). PCR mixes (total volume: 14 μl) included 8.5 μl ultrapure water, 1.25 μl PCR buffer, 1.15 μl MgCl_2_ (50 mM), 0.25 μl of each primer (10 μM) (forward: L14724: 5′-CGAAGCTTGATATGAAAAACCATCGTTG-3′, reverse: H15149: 5′-AAACTGCAGCCCCTCAGAATGATATTTGTCCTCA-3′^47^,^99^), 0.02 μl of each dNTP (12.5 μM), 0.8 μl Taq (5 U/μl) polymerase and 1.0 μl DNA template. Thermocycling conditions were as follows: initial denaturation for 3 min at 95 °C, followed by 35 cycles of denaturation at 95 °C for 40 s, primer annealing at 52 °C for 40 s, extension at 72 °C for 30 s, and a final extension step at 72 °C for 5 min. PCR products were cleaned using the Bioline SureClean kit according to the manufacturer’s protocol. Sequencing was outsourced to GATC Biotech AG (Konstanz, Germany). We sequenced fragments in both directions.

Sequences were checked for stop codons and aligned manually (as there were no insertions/deletions that might have caused ambiguous alignment alternatives), including reference sequences and sequences of closely related *Gambusia* spp. from Genbank, as well as one sequence of *Belonesox belizanus* that served as an outgroup^47^. Bayesian phylogenetic inference was conducted in MrBayes 3.2.2^100^ under a GTR + G substitution model with four chains and two independent runs for 7M iterations, sampling every 5,000^th^ iteration. Potential autocorrelation (effective sample size for all parameters >950) and stationarity of the Marcov chain were checked in Tracer 1.6^101^. A maximum clade credibility tree (Fig. 3B) was calculated in TreeAnnotator 1.8.2 (part of the BEAST package^102^), with a burn-in of 350 trees.

#### Population genetic analysis

We used 15 nuclear microsatellite loci to genotype *n* = 239 fish from all 10 sites (Table 3; Fig. 3A). This allowed verification of species identity and testing for potential signs of hybridization by using a population genetic approach. We used primer pairs established for *G. affinis*^103^,^104^, which were arranged in three separate multiplex reactions (*reaction 1*: Gaaf10, Gaaf11, Gaaf13, Gafμ3; *reaction 2*: Gaaf7, Gaaf9, Gaaf15, Gaaf16, Gaaf22, Gafμ2, Gafμ6; *reaction 3*: Gafμ1, Gafμ4, Gafμ7, Mf13). Microsatellites were amplified with the Type-it Microsatellite PCR kit from Qiagen (Hilden, Germany). The PCR protocol included an initial denaturation step for 5 min at 95 °C, 28 cycles at 95 °C for 30 s (denaturation), 57 °C for 90 s (primer annealing), and 72 °C for 30 s (extension), followed by a final extension step for 30:00 min at 60 °C. The 5 μl reaction mix included 2.5 μl Type-it master mix, 0.4 μl primer mix, 0.4 μl Q-solution, 0.9 μl RNase-free water, and 0.8 μl template DNA. PCR products were analysed on a CEQ2000 sequencer (Beckman Coulter; denaturation at 90 °C for 2 min, injection at 2.0 kV for 30 s, separation at 6.0 kV for 45 min) along with the manufacturer’s internal size standard. We screened the resulting fragment length data using Genome Lab GeTX 10.2 software (Beckman Coulter) and performed allele-calling manually.

We used the software STRUCTURE 2.3.4^105^ to calculate individual assignment probabilities (*Q*-values) to varying numbers of genetically distinct clusters (*K*). For each value of *K* = 1–12, ten iterations were run using the admixture model with a burn-in period of 250,000 generations, followed by a sampling phase of 750,000 iterations. We detected the uppermost level of population differentiation with the method presented by Evanno *et al*.^64^ using the web-based tool STRUCTURE HARVESTER 0.6.93^106^. To detect potential hybrids, we used the software NEWHYBRIDS^107^. This approach also provided *Q*-values, which describe the probability that an individual belongs to each of six different genotypes (i.e., parental ‘purebreds’, F_1_-hybrids, F_2_-hybrids and backcrosses with either parental genotype). We ran NEWHYBRIDS with a burn-in period of 250,000 generations, followed by a sampling phase of 750,000 iterations. We considered individuals to be ‘purebreds’ if *Q* ≥ 0.95.

To calculate standard indicators of genetic variability and pairwise *F*_ST_-values between populations, we conducted species-wise analyses, thus excluding single individuals of the respective other species if a site was dominated by one of the two *Gambusia* species. For example, in case of site 2 we excluded the single individual that was identified as *G. yucatana*. However, in the case of site 5, where both species occurred at sufficiently high frequencies, we analysed the subset of *G. sexradiata* (*n* = 5) and *G. yucatana* (*n* = 19), separately. We used ARLEQUIN v 3.5^108^ to calculate pairwise *F*_ST_-values and used FSTAT^109^ to calculate standard population genetic metrics, namely observed (*H*_O_) and expected heterozygosity (*H*_E_) as well as allelic richness (*A*) and to conduct a probability test for deviations from Hardy-Weinberg equilibrium (HWE).

### Species distributions patterns

We used canonical correspondence analysis CCA^110^, to analyse patterns of variation of fish assemblage composition along our sample sites in the Río Grijalva basin in relation to environmental characteristics and to evaluate whether and how environmental variables explain the occurrence of both congeneric *Gambusia* species. The first CCA was conducted using presence/absence data of all species as the dependent data matrix, and the three environmental PCs (see above) as independent variables. For the second CCA we used numbers of both *Gambusia* species at each site (obtained from seining) as the dependent data matrix, and the three environmental PCs as independent variables. Both CCAs were conducted with a Monte Carlo permutation test using XLSTAT 2016^111^.

### Phenotypic divergence

#### Geometric morphometric analyses

One multivariate measure of phenotypic trait divergence along the stream gradient was body shape variation, which we assessed for 32 to 40 individuals per population (*n* = 384 individuals altogether; Table 3) using landmark-based geometric morphometric analysis. Photographs were taken in lateral view using a Canon eos 600D camera mounted on a stand. We digitized 15 lateral landmark coordinates using the software program tpsDig2^112^. Landmarks were selected to provide adequate coverage of the lateral body profile and largely followed previous studies on *Gambusia* spp.^33^,^113^ with additional landmark points defined at the eyes and pectoral fins (see Supplementary Fig. 2). To account for bending of specimens owing to preservation, we unbent landmarks using the landmarks at the tip of the mouth and middle of the tail fin and two additional temporary landmarks set at the lateral line (but removed in the final analyses) using the ‘unbend specimens’ function in tpsUtil^114^. Procrustes fits were obtained using a full Procrustes fits procedure implemented in the software morphoJ^115^ that orthogonally projects landmark data to a tangent space and automatically excludes variation that is not caused by true shape-variation (e.g., differences due to scaling and positioning of the test subjects; for a detailed description of geometric morphometrics see ref. 116). We subjected procrustes coordinates to a factor reduction and retained the first 5 relative warps (RWs), which are principal components of shape variation, explaining 73.6% (males) and 70.3% (females) of the morphological variance, respectively. We analysed males and females separately since mature *Gambusia* species show pronounced sexual dimorphism in an array of morphological traits^75^.

We tested our predictions regarding shared and unique patterns of gradient evolution (Fig. 2) by assessing the relative contributions of species identity (reflecting a phylogenetic signal) and environmental conditions at each sample site to shape variation. We used the ‘morphological PCs’ as dependent variables in two multivariate GLMs (MANCOVAs, one for each sex), in which ‘species’ was specified as fixed factor. Log-transformed ‘body centroid size’ and the three ‘environmental PCs’ were included as covariates. We included all two-way interactions of covariates with the fixed factor. Inspection of model residuals did not indicate violations of model assumptions (i.e., normal error distribution and homoscedasticity). To quantify the relative importance of model terms, we estimated effect sizes using Wilk’s partial eta squared (*η*_p_^2^) and calculated relative variances as the partial variance for a given term divided by the maximum partial variance value in that model.

#### Life history measurements

Dissections to collect male, female, and offspring-related life-history traits followed well established protocols^21^,^88^. We collected the following male and female life-history traits from 21 to 38 individuals per population (*n* = 301 altogether; Table 3): standard length (SL [mm]), dry weight [g], lean weight [g], fat content [%], and reproductive investment ([%]; for males: testis dry weight divided by the sum of reproductive and somatic tissue dry weight, i.e., gonadosomatic index (GSI); for females: offspring dry weight divided by the sum of offspring plus somatic dry weight, i.e., reproductive allocation (RA)). In case of females, we further determined offspring lean weight [mg], offspring fat content [%] and fecundity (number of offspring per clutch). During dissections performed on gravid females, all developing offspring were removed and their stage of development determined according to the classification scheme outlined in Riesch *et al*.^117^.

As predicted^21^,^66^,^88^, a preparatory GLM revealed an effect of embryo stage on embryo lean weight, and so we used residuals for further analysis. Prior to statistical analyses we log_10_-transformed (male and female SL, male and female lean weight, and embryo lean weight), square root-transformed (fecundity), or arcsine (square root)-transformed (male, female, and embryo fat content, male GSI and female RA) all life-history variables, and used subsequent *z*-transformation to meet assumptions of statistical analyses (i.e., these transformations greatly facilitated normality of model residuals).

To evaluate the mode of maternal provisioning, we calculated the matrotrophy index (MI) using the slopes and intercepts from linear regressions by analysing the relationship between log-transformed embryonic dry mass and stage of development^65^,^66^. If the eggs were fully provisioned by yolk before fertilization (lecithotrophy), then we would expect the embryos to lose 25–40% of their dry mass during development (MI between 0.6 and 0.7; see ref. 118). On the other hand, in the case of continuous maternal provisioning after fertilization (matrotrophy), one would expect the embryos to lose less weight (MI between 0.7 and 1.0) or to even gain weight during development (MI > 1.00; see e.g., ref. 65).

We excluded site 5 and 8 from the analyses of maternal provisioning due to a low number of pregnant females of each species (*n* ≤ 4). We calculated the MI for species separately, that means in the case of site 3, we used the subset of *G. sexradiata* (*n* = 16) and omitted pregnant females of *G. yucatana* (*n* = 2). We tested for differences in MI under different environmental conditions in a GLM, in which we included ‘MI’ as the dependent variable, ‘environmental PCs’ as covariates, and ‘species’ a fixed factor. We then tested each population separately for significant divergence from an MI of 0.7 (a cutoff-point, with values below being indicative of lecithotrophy and values above indicating at least some level of incipient matrotrophy). We tested each population against that population’s hypothetical slope for an MI of 0.7 using one-sample *z*-tests (following Reznick *et al*.^65^).

The main statistical analysis of life-history traits followed our approach described for the morphometric analysis (see above). We used the *z*-transformed life-history variables as dependent variables in multivariate GLMs for both sexes separately, with ‘species’ specified as fixed factor, and ‘SL’ and ‘environmental PCs’ as covariates. Again, we included all two-way interactions of covariates with the fixed factor. The evaluate differences among populations in body size, we conducted a univariate GLM for both sexes combined, in which we used log transformed ‘SL’ as the dependent variable, ‘species’ and ‘sex’ as fixed factors, and ‘environmental PCs’ as covariates. Inspection of model residuals did not indicate violations of model assumptions (i.e., normal error distribution and homoscedasticity) in all models described in this article.

To test for a potential effect of neutral genetic population differentiation on phenotypic differences among populations, we focussed on *G. sexradiata*, since we found no genetic structure within *G. yucatana*. To summarise phenotypic divergence we conducted a PCA using all RWs and life history parameters; in case of a significant influence of SL (assessed via Pearson correlations), we used residuals corrected for SL. Based on the retained five (males) and six (females) PCs with an eigenvalue >1.0 (Supplementary Tables S9 and S10), we calculated Euclidean distances across populations and compared these with *F*_ST_-values by conducting a partial Mantel test using the software PASSAGE 2.0.11.6.

## Additional Information

**How to cite this article**: Jourdan, J. *et al*. Shared and unique patterns of phenotypic diversification along a stream gradient in two congeneric species. *Sci. Rep.* **6**, 38971; doi: 10.1038/srep38971 (2016).

**Publisher’s note:** Springer Nature remains neutral with regard to jurisdictional claims in published maps and institutional affiliations.

## Supplementary Material

Supplementary Information

## Figures and Tables

**Table 1:** Analyses of phenotypic trait divergence.

Model	Source	*F*	d.f.	*P*	Partial variance explained (%)
(*a*) male body shape	Species	1.778	5, 172	0.120	14.31
	**Centroid size**	**17.903**	**5, 172**	<**0.001**	**99.69**
	**Environmental PC 1**	**3.126**	**5, 172**	**0.010**	**24.26**
	Environmental PC 2	0.905	5, 172	0.479	7.46
	Environmental PC 3	1.547	5, 172	0.178	12.53
	Species × Centroid size	1.924	5, 172	0.093	15.43
	**Species** × **Environmental PC 1**	**2.935**	**5, 172**	**0.014**	**22.89**
	Species × Environmental PC 2	1.623	5, 172	0.156	13.12
	**Species** × **Environmental PC 3**	**3.773**	**5, 172**	**0.003**	**28.79**
(*b*) female body shape	**Species**	**2.387**	**10, 168**	**0.040**	**16.56**
	**Centroid size**	**20.208**	**10, 168**	<**0.001**	**96.34**
	Environmental PC 1	1.722	10, 168	0.132	12.15
	**Environmental PC 2**	**8.396**	**10, 168**	<**0.001**	**50.49**
	**Environmental PC 3**	**6.917**	**10, 168**	<**0.001**	**43.00**
	Species × Centroid size	2.245	10, 168	0.052	15.63
	**Species** × **Environmental PC 1**	**3.094**	**10, 168**	**0.010**	**21.08**
	Species × Environmental PC 2	1.033	10, 168	0.400	7.42
	**Species** × **Environmental PC 3**	**2.520**	**10, 168**	**0.031**	**17.42**
(*c*) male life histories	**Species**	**4.542**	**3, 161**	**0.004**	**8.90**
	**Length**	**379.008**	**3, 161**	<**0.001**	**100.00**
	**Environmental PC 1**	**14.791**	**3, 161**	<**0.001**	**24.66**
	Environmental PC 2	1.311	3, 161	0.273	2.74
	**Environmental PC 3**	**4.674**	**3, 161**	**0.004**	**9.13**
	Species × Length	1.053	3, 161	0.371	2.17
	**Species** × **Environmental PC 1**	**11.419**	**3, 161**	<**0.001**	**19.98**
	**Species** × **Environmental PC 2**	**4.862**	**3, 161**	**0.003**	**9.47**
	**Species** × **Environmental PC 3**	**3.022**	**3, 161**	**0.031**	**6.05**
(*d*) female life histories	**Species**	**2.366**	**6, 113**	**0.034**	**13.58**
	**Length**	**88.980**	**6, 113**	<**0.001**	**100.00**
	**Environmental PC 1**	**3.062**	**6, 113**	**0.008**	**16.97**
	**Environmental PC 2**	**8.125**	**6, 113**	<**0.001**	**36.48**
	**Environmental PC 3**	**2.264**	**6, 113**	**0.042**	**12.97**
	Species × Length	0.685	**6, 113**	0.662	4.24
	**Species** × **Environmental PC 1**	**2.820**	**6, 113**	**0.014**	**15.76**
	**Species** × **Environmental PC 2**	**2.603**	**6, 113**	**0.021**	**14.67**
	Species × Environmental PC 3	2.136	6, 113	0.055	12.36
(*e*) SL both sexes	**Species**	**23.292**	**1, 289**	<**0.001**	**7.72**
	**Sex**	**119.811**	**1, 289**	<**0.001**	**72.90**
	Environmental PC 1	3.724	1, 289	0.055	3.16
	**Environmental PC 2**	**46.357**	**1, 289**	**0.000**	**34.39**
	Environmental PC 3	1.814	1, 289	0.179	1.55
	Species × Sex	2.933	1, 289	0.088	2.50
	**Sex** × **Environmental PC 1**	**5.146**	**1, 289**	**0.024**	**4.35**
	Sex × Environmental PC 2	3.340	1, 289	0.069	2.84
	**Sex** × **Environmental PC 3**	**4.257**	**1, 289**	**0.040**	**3.61**
	Species × Environmental PC 1	0.105	1, 289	0.747	0.09
	Species × Environmental PC 2	0.004	1, 289	0.948	0.00
	Species × Environmental PC 3	2.971	1, 289	0.086	2.53

Results of MANCOVAs examining (*a*) female and (*b*) male shape variation as well as (*c*) female and (*d*) male life history differentiation along environmental gradients. (*e*) Results of ANCOVA examining body length in both sexes. *F* ratios were approximated using Wilk’s λ values. Partial variance explained was estimated using Wilk’s partial η^2^ (for details see main text).

**Table 2:** Results of principal component analysis of the 12 environmental variables.

	Environmental PC		
	1	2	3
Predation risk	0.196	**0.862**	−0.378
Water depth [m]	0.190	**0.934**	0.037
pH	0.581	**0.722**	0.151
DO [%]	0.047	**0.872**	0.226
Conductivity [μS/cm]	0.091	**0.628**	−**0.607**
Salinity [ppt]	0.056	**0.635**	−**0.611**
Mean annual temperature [°C]	**0.829**	0.229	0.453
Max. temperature of the warmest month [°C]	0.088	0.099	**0.928**
Temperature of the coldest month [°C]	**0.977**	0.131	−0.137
Annual temperature range [°C]	−0.444	0.014	**0.852**
Annual precipitation [mm]	−**0.951**	−0.167	0.164
Altitude [m]	−**0.919**	−0.103	0.249

Environmental conditions were measured at ten study sites located within the Río Grijalva basin. PC loadings ≥|0.6| are shown in bold type.

**Table 3:** Overview of sample sites.

Site ID	Site name	Latitude, longitude	*n* population genetics	*n* body shape	*n* life history
1	Laguna Mecoacán	18.342, −93.125	12/12	20/20	28/9
2	Simon Sarlat	18.337, −92.779	12/12	17/20	14/17
3	Laguna de Las Ilusiones	18.019, −92.931	12/12	20/20	20/18
4	Laguna Ranchería 1^ra^ Lázaro Cárdenas	18.003, −93.022	12/12	20/20	19/18
5	Campus DACBiol-UJAT	17.989, −92.975	12/12	17/17	15/8
6	Ismate Chilapilla	17.913, −92.545	12/12	20/20	18/17
7	San Antonio I	17.749, −92.896	12/12	14/20	9/13
8	San Antonio II	17.734, −92.878	12/11	18/21	16/5
9	Laguna Canto Rodado	17.589, −92.981	12/12	20/20	15/19
10	Pond near Teapa	17.556, −92.952	12/12	20/20	19/15

Site locations and numbers of individuals (*n*; males/females) used for the morphometric, life-history and population genetic analyses.
