# Supplementary material for: Shared and unique patterns of phenotypic diversification along a stream gradient in two congeneric species
Source: Sci Rep. 2016 Dec 16;6:38971. doi: 10.1038/srep38971 (PMC5159898; doi:10.1038/srep38971)
Supplement: Supplementary Information [file srep38971-s1.pdf]

Supplementary Information

Shared and unique patterns of phenotypic diversification along a stream gradient in two congeneric species

Jonas Jourdan[1,2,3*], Sarah T. Krause[2], V. Max Lazar[2], Claudia Zimmer[1,2], Carolin Sommer-Trembo[2], Lenin Arias-Rodriguez[4], Sebastian Klaus[2], Rüdiger Riesch[5] and Martin Plath[1]

[1]*College of Animal Science and Technology, Northwest A&F University, Yangling, Shaanxi 712100, P.R. China*

[2]*Goethe University of Frankfurt, Department of Ecology and Evolution, Max-von-Laue-Straße 13, D-60438 Frankfurt am Main, Germany*

[3]*Department of River Ecology and Conservation, Senckenberg Research Institute and Natural History Museum Frankfurt, Gelnhausen, Germany*

[4]*División Académica de Ciencias Biológicas, Universidad Juárez Autónoma de Tabasco (UJAT), C.P. 86150 Villahermosa, Tabasco, México*

[5]*School of Biological Sciences, Royal Holloway University of London, Egham, Surrey, TW20 0EX, UK*

Environmental conditions

Table S1. Spatial variation of environmental factors among the ten sample sites.

Site ID	pH	Dissolved oxygen [mg/L]	Dissolved oxygen [%]	Conductivity [µs/cm]	Dissolved salt concentration [ppt]	Mean annual temperature [°C]	Predation risk	water depth [m]
1	8.25	7.79	112.5	8700	7	26.4	high	> 5
2	7.40	1.19	16.5	540	0	26.2	medium	1-3
3	9.20	10.67	161.0	209	0	27.0	medium	> 5
4	8.05	7.64	112.2	482	0	26.9	medium	3-5
5	6.92	0.59	7.8	757	0	26.9	low	< 1
6	7.34	3.76	52.4	175	0	26.4	low	< 1
7	7.22	0.41	5.3	350	0	26.4	low	1-3
8	6.61	0.30	3.7	256	0	26.4	low	1-3
9	7.94	8.24	115.2	441	0	26.1	medium	3-5
10	5.44	3.70	47.6	72	0	25.7	low	< 1

Table S2. Summary information on fish communities at the ten sample sites.

Site ID	Site name	Predation risk category	Accompanying fauna
1	Laguna Mecoacán	High	*Atherinella alvarezi* (Atherinopsidae)<br>*Centropomus undecimalis* (Centropomidae)<br>*Anchoa mitchilli* (Engraulidae)<br>*Dormitator maculatus* (Eleotridae)<br>*Belonesox belizanus* (Poeciliidae)<br>*Poecilia mexicana* (Poeciliidae)<br>*Poecilia sphenops* (Poeciliidae)<br>*Microphis brachyurus* (Syngnathidae)<br>Anguillidae indet., larvae<br>Cichlidae indet.<br>Goobidae indet.
2	Simon Sarlat	Medium	*Dormitator maculatus* (Eleotridae)<br>*Belonesox belizanus* (Poeciliidae)<br>*Phallichthys fairweatheri* (Poeciliidae)<br>Cichlidae indet.
3	Laguna de Las Ilusiones	Medium	*Atherinella alvarezi* (Atherinopsidae)<br>*Astyanax aeneus* (Characidae)<br>*Thorichthys passionis* (Cichlidae)<br>*Dorosoma* sp. (Clupeidae)<br>*Pterygoplichthys* sp. (Loricariidae)<br>*Belonesox belizanus* (Poeciliidae)<br>*Poecilia mexicana* (Poeciliidae)<br>Cichlidae indet.
4	Laguna Ranchería 1ra Lázaro Cárdenas	Medium	*Atherinella alvarezi* (Atherinopsidae)<br>*Pterygoplichthys* sp. (Loricariidae)<br>*Belonesox belizanus* (Poeciliidae)<br>*Xiphophorus maculatus* (Poeciliidae)<br>*Cynodonichthys tenuis* (Rivulidae)
5	Campus DACBiol-UJAT	Low	*Pseudoxiphophorus bimaculatus* (Poeciliidae)<br>*Xiphophorus maculatus* (Poeciliidae)<br>*Cynodonichthys tenuis* (Rivulidae)
6	Ismate Chilapilla	Low	*Rhamdia* cf. *guatemalensis* (Heptapteridae)<br>*Xiphophorus maculatus* (Poeciliidae)<br>*Cynodonichthys tenuis* (Rivulidae)
7	San Antonio I	Low	*Astyanax aeneus* (Characidae)<br>*Poecilia petenensis* (Poeciliidae)<br>*Xiphophorus maculatus* (Poeciliidae)<br>*Cynodonichthys tenuis* (Rivulidae)
8	San Antonio II	Low	*Astyanax aeneus* (Characidae)<br>*Poecilia petenensis* (Poeciliidae)<br>*Xiphophorus maculatus* (Poeciliidae)<br>*Cynodonichthys tenuis* (Rivulidae)
9	Laguna Canto Rodado	Medium	*Pterygoplichthys* sp. (Loricariidae)<br>*Belonesox belizanus* (Poeciliidae)<br>Cichlidae indet.
10	Pond near Teapa	low	*Pseudoxiphophorus bimaculatus* (Poeciliidae)<br>*Xiphophorus maculatus* (Poeciliidae)<br>*Cynodonichthys tenuis* (Rivulidae)

Population genetic analyses

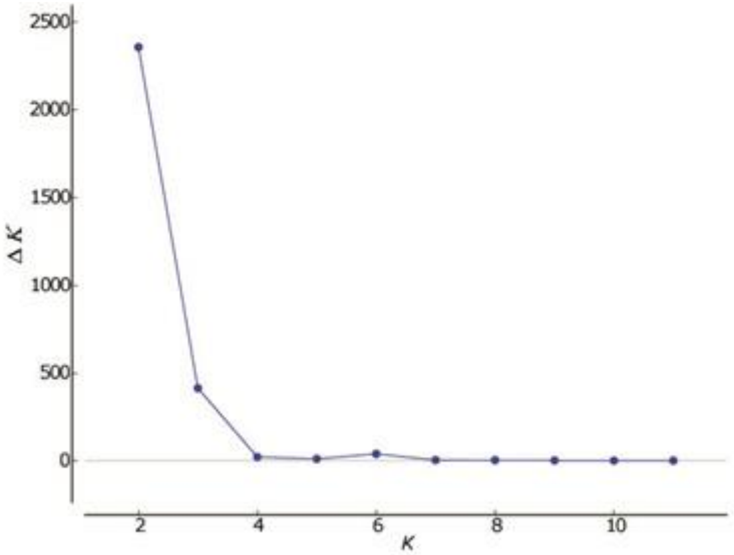

Figure S1. Bayesian inference of the number of genetically distinct clusters (*K*) among the 10 sampled populations using Δ*K* (Evanno *et al*. 2005).

Table S3. Genetic differentiation (estimated by pairwise F_{ST}) among study sites. To calculate F_{ST}-values we removed individuals which did not assign to the major cluster found at each site except study site 5, which was divided into a *G. yucatana* (5_*yuc*.) and a *G. sexradiata* (5_*sex*.) group. Significant ($P < 0.05$) F_{ST}-values are given in bold typeface.

	**1**	**2**	**3**	**4**	**5_*yuc.***	**5_*sex.***	**6**	**7**	**8**	**9**	**10**
**1**	0										
**2**	**0.337**	0									
**3**	**0.373**	0.032	0								
**4**	**0.372**	0.036	0.006	0							
**5_*yuc.***	0.023	**0.365**	**0.403**	**0.396**	0						
**5_*sex.***	**0.430**	**0.069**	0.032	0.032	**0.470**	0					
**6**	**0.354**	0.008	**0.066**	**0.066**	**0.381**	**0.082**	0				
**7**	**0.335**	0.017	0.014	0.035	**0.361**	0.050	0.043	0			
**8**	0.034	**0.351**	**0.388**	**0.386**	0.044	**0.450**	**0.368**	**0.346**	0		
**9**	**0.386**	0.049	0.041	0.043	**0.409**	**0.088**	**0.069**	0.039	**0.392**	0	
**10**	**0.461**	**0.214**	**0.223**	**0.220**	**0.488**	**0.252**	**0.223**	**0.210**	**0.484**	**0.271**	0

Table S4. For each population and locus, observed (H_O) and expected (H_E) heterozygosities as well as allelic richness (A) are given. Zero values indicate that the locus is monomorphic in this population. Ranges of allele sizes are given for the entire data set.

**Locus**	**Number of alleles**	**Range of allele size**	**Test**	**1** n = 24	**2** n = 23	**3** n = 21	**4** n = 24	**5_*yuc*** n = 19	**5_*sex*** n = 5	**6** n = 24	**7** n = 24	**8** n = 23	**9** n = 24	**10** n = 24	**Mean across populations**
**Gaaf10**	17	218-282	H_O	0.22	0.67	0.53	0.65	0.10	0.40	0.55	0.43	0.14	0.57	0.39	0.43
			H_E	0.34	0.80	0.83	0.73	0.81	0.64	0.86	0.81	0.82	0.72	0.39	0.74
			A	4.34	4.67	4.82	4.15	4.57	3.58	5.01	4.51	4.45	3.69	2.17	4.18
**Gaaf11**	23	87-187	H_O	0.18	0.80	0.71	0.58	0.38	0.80	0.91	0.88	0.35	0.35	0.38	0.57
			H_E	0.17	0.87	0.76	0.65	0.33	0.84	0.90	0.83	0.30	0.47	0.51	0.60
			A	1.57	5.51	4.57	3.97	2.18	5.18	5.99	5.06	1.94	2.78	2.95	3.79
**Gaaf13**	49	111-327	H_O	0.17	0.82	0.84	0.87	0.06	0.40	0.47	0.75	0.00	0.88	0.48	0.52
			H_E	0.17	0.97	0.97	0.97	0.06	0.82	0.96	0.96	0.00	0.94	0.45	0.66
			A	1.70	7.10	7.11	7.12	1.25	4.56	7.04	7.07	1.00	6.56	2.86	4.85
**Gafμ3**	39	145-285	H_O	0.75	0.74	0.89	0.83	0.82	0.80	0.83	0.88	0.74	1.00	0.67	0.81
			H_E	0.89	0.95	0.96	0.93	0.89	0.80	0.93	0.95	0.89	0.94	0.79	0.90
			A	5.52	6.78	6.96	6.31	5.55	4.40	6.51	6.85	5.64	6.57	4.20	5.94
**Gaaf7**	6	125-141	H_O	0.22	0.05	0.10	0.00	0.28	0.00	0.04	0.21	0.52	0.04	0.00	0.13
			H_E	0.34	0.05	0.10	0.00	0.25	0.00	0.13	0.20	0.52	0.04	0.00	0.15
			A	2.20	1.20	1.40	1.00	1.88	1.00	1.50	1.74	2.60	1.17	1.00	1.52
**Gaaf9**	2	224-228	H_O	0.00	0.00	0.00	0.00	0.00	0.00	0.00	0.00	0.00	0.00	0.00	0.00
			H_E	0.09	0.00	0.00	0.09	0.00	0.00	0.00	0.00	0.00	0.00	0.00	0.02
			A	1.32	1.00	1.00	1.33	1.00	1.00	1.00	1.00	1.00	1.00	1.00	1.06
**Gaaf15**	7	130-154	H_O	0.39	0.50	0.45	0.45	0.50	0.80	0.43	0.42	0.45	0.63	0.30	0.48
			H_E	0.64	0.59	0.52	0.60	0.61	0.69	0.42	0.56	0.46	0.64	0.26	0.54
			A	3.47	3.00	2.19	2.78	3.48	2.98	2.30	2.46	2.80	2.78	1.76	2.73
**Gaaf16**	14	207-279	H_O	0.62	0.95	0.60	0.83	0.37	0.80	0.91	0.67	0.67	0.74	0.35	0.68
			H_E	0.59	0.83	0.82	0.85	0.47	0.80	0.85	0.68	0.75	0.65	0.49	0.71
			A	3.00	4.65	4.71	5.00	2.64	4.40	5.14	3.62	3.87	3.56	2.83	3.95
**Gaaf22**	41	211-388	H_O	1.00	1.00	0.80	0.91	0.74	0.40	0.96	0.91	0.95	1.00	0.50	0.83
			H_E	0.91	0.96	0.94	0.94	0.80	0.67	0.94	0.95	0.89	0.94	0.65	0.87
			A	5.97	6.96	6.62	6.50	4.88	4.20	6.49	6.72	5.78	6.64	3.54	5.84
**Gafμ2**	24	110-186	H_O	0.17	0.86	0.50	0.96	0.16	0.60	0.83	0.67	0.18	0.83	0.35	0.56
			H_E	0.17	0.91	0.79	0.87	0.20	0.78	0.91	0.85	0.22	0.86	0.45	0.64
			A	1.62	6.05	4.64	5.40	1.84	3.78	5.92	4.96	1.91	4.98	1.97	3.92
**Gafμ6**	15	137-205	H_O	0.13	0.50	0.05	0.17	0.16	0.00	0.48	0.13	0.20	0.43	0.45	0.25
			H_E	0.79	0.75	0.14	0.27	0.60	0.00	0.79	0.49	0.70	0.60	0.61	0.52
			A	4.36	4.03	1.50	1.98	3.27	1.00	4.25	2.63	3.46	3.24	2.87	2.96
**Gafμ1**	8	103-119	H_O	0.09	0.18	0.05	0.00	0.00	0.00	0.30	0.00	0.04	0.08	0.00	0.07
			H_E	0.20	0.62	0.49	0.39	0.11	0.36	0.62	0.49	0.04	0.54	0.31	0.38
			A	1.79	2.71	2.16	1.93	1.42	1.98	2.74	2.28	1.17	2.30	2.03	2.05
**Gafμ4**	17	173-199	H_O	0.61	0.14	0.43	0.30	0.79	0.50	0.25	0.17	0.65	0.00	0.42	0.39
			H_E	0.77	0.13	0.36	0.37	0.80	0.43	0.23	0.41	0.83	0.00	0.51	0.44
			A	4.32	1.52	2.15	2.29	4.26	2.00	1.78	2.33	4.62	1.00	2.16	2.58
**Gafμ7**	34	155-237	H_O	0.83	0.82	0.81	0.87	0.50	1.00	0.75	0.67	0.74	0.48	0.25	0.70
			H_E	0.87	0.94	0.92	0.94	0.76	0.93	0.91	0.94	0.83	0.87	0.45	0.85
			A	5.27	6.57	6.31	6.45	3.84	6.00	5.98	6.52	4.67	5.51	2.65	5.43
**Mf-13**	17	165-191	H_O	0.29	0.73	0.62	0.67	0.26	0.50	0.79	0.75	0.18	0.67	0.58	0.55
			H_E	0.45	0.85	0.74	0.74	0.46	0.68	0.83	0.76	0.18	0.69	0.68	0.64
			A	2.76	5.00	3.84	3.74	2.91	3.00	4.72	4.01	1.73	3.57	3.28	3.50
**Mean across loci**			H_O	0.38	0.58	0.49	0.54	0.34	0.47	0.57	0.50	0.39	0.51	0.34	
			H_E	0.52	0.68	0.62	0.62	0.48	0.56	0.68	0.66	0.50	0.59	0.44	
			A	3.28	4.45	4.00	4.00	3.00	3.27	4.42	4.12	3.11	3.69	2.48	

Geometric morphometric analyses

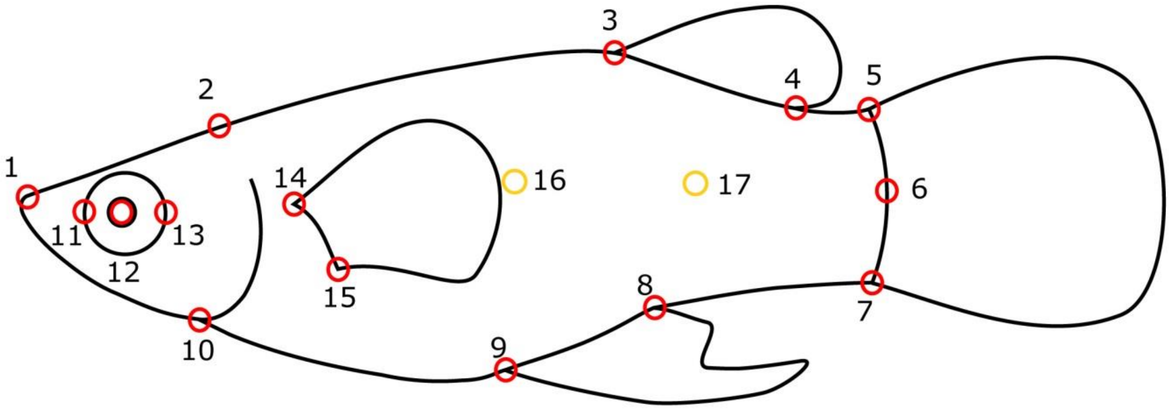

Figure S2. The 15 landmarks for the morphological analyses included (1) the tip of the upper jaw, (2) the posterodorsal tip of the supraoccipital crest, (3) the anterior and (4) posterior insertions of the dorsal fin, (5) the dorsal, (6) central and (7) ventral insertions of the caudal fin, (8) the posterior and (9) anterior junctions of the anal fin, (10) the bottom of the head where the operculum breaks away from the body outline, (11) the anterior, (12) centre and posterior (13) margins of the eye, as well as (14) the dorsal and (14) ventral insertions of the pectoral fin. Two additional temporary landmarks (16, 17) were placed at the lateral line and used to account for bending of specimens owing to preservation (but removed in the final analyses).

Patterns of phenotypic diversification

Table S5. Results from ANCOVAs on morphometric variables (relative warps, RW) using ‘species’ as a fixed factor, and ‘centroid size’ and environmental variables (three PCs) as covariates.

Source	Dependent variable	d.f.	*F*	*p*	Relative variance explained (%)
Species	Male RW 1	1, 176	1.244	0.266	5.74
	Male RW 2	1, 176	0.192	0.662	0.89
	Male RW 3	1, 176	6.662	0.011	29.82
	Male RW 4	1, 176	0.297	0.586	1.38
	Male RW 5	1, 176	1.032	0.311	4.77
Centroid size	Male RW 1	1, 176	14.873	< 0.001	63.71
	Male RW 2	1, 176	0.674	0.413	3.12
	Male RW 3	1, 176	7.537	0.007	33.58
	Male RW 4	1, 176	24.525	< 0.001	100.00
	Male RW 5	1, 176	7.096	0.008	31.69
Environmental PC 1	Male RW 1	1, 176	6.165	0.014	27.67
	Male RW 2	1, 176	0.711	0.400	3.29
	Male RW 3	1, 176	6.039	0.015	27.12
	Male RW 4	1, 176	2.063	0.153	9.47
	Male RW 5	1, 176	1.699	0.194	7.82
Environmental PC 2	Male RW 1	1, 176	0.954	0.330	4.41
	Male RW 2	1, 176	0.761	0.384	3.52
	Male RW 3	1, 176	0.088	0.767	0.41
	Male RW 4	1, 176	3.302	0.071	15.06
	Male RW 5	1, 176	0.355	0.552	1.65
Environmental PC 3	Male RW 1	1, 176	4.267	0.040	19.36
	Male RW 2	1, 176	0.002	0.968	0.01
	Male RW 3	1, 176	1.714	0.192	7.89
	Male RW 4	1, 176	1.224	0.270	5.65
	Male RW 5	1, 176	1.084	0.299	5.01
Species × Centroid size	Male RW 1	1, 176	1.536	0.217	7.08
	Male RW 2	1, 176	0.017	0.897	0.08
	Male RW 3	1, 176	7.616	0.006	33.91
	Male RW 4	1, 176	0.528	0.468	2.45
	Male RW 5	1, 176	0.826	0.365	3.82
Species × Environmental PC 1	Male RW 1	1, 176	5.400	0.021	24.34
	Male RW 2	1, 176	0.608	0.437	2.81
	Male RW 3	1, 176	7.858	0.006	34.94
	Male RW 4	1, 176	1.464	0.228	6.75
	Male RW 5	1, 176	0.063	0.802	0.29
Species × Environmental PC 2	Male RW 1	1, 176	2.815	0.095	12.87
	Male RW 2	1, 176	0.237	0.627	1.10
	Male RW 3	1, 176	0.876	0.351	4.05
	Male RW 4	1, 176	2.613	0.108	11.96
	Male RW 5	1, 176	1.551	0.215	7.14
Species × Environmental PC 3	Male RW 1	1, 176	2.913	0.090	13.31
	Male RW 2	1, 176	5.267	0.023	23.76
	Male RW 3	1, 176	5.577	0.019	25.11
	Male RW 4	1, 176	2.849	0.093	13.02
	Male RW 5	1, 176	0.003	0.960	0.01

Table S6. Results from ANCOVAs on morphometric variables of females (RW 1 through 5) using ‘species’ as a fixed factor, and ‘centroid size’ and environmental variables (PC 1 through 3) as covariates.

Source	Dependent variable	d.f.	*F*	*p*	Relative variance explained (%)
Species	Female RW 1	1, 188	6.720	0.010	11.27
	Female RW 2	1, 188	3.078	0.081	5.26
	Female RW 3	1, 188	1.744	0.188	3.00
	Female RW 4	1, 188	2.367	0.126	4.06
	Female RW 5	1, 188	0.664	0.416	1.15
Centroid size	Female RW 1	1, 188	78.629	< 0.001	96.31
	Female RW 2	1, 188	6.093	0.014	10.25
	Female RW 3	1, 188	3.287	0.071	5.61
	Female RW 4	1, 188	0.105	0.747	0.18
	Female RW 5	1, 188	3.333	0.070	5.69
Environmental PC 1	Female RW 1	1, 188	2.439	0.120	4.18
	Female RW 2	1, 188	1.323	0.252	2.28
	Female RW 3	1, 188	0.428	0.514	0.74
	Female RW 4	1, 188	4.196	0.042	7.13
	Female RW 5	1, 188	0.741	0.391	1.28
Environmental PC 2	Female RW 1	1, 188	15.919	< 0.001	25.49
	Female RW 2	1, 188	5.644	0.019	9.52
	Female RW 3	1, 188	3.425	0.066	5.84
	Female RW 4	1, 188	0.530	0.468	0.92
	Female RW 5	1, 188	0.013	0.909	0.02
Environmental PC 3	Female RW 1	1, 188	15.903	< 0.001	25.47
	Female RW 2	1, 188	3.121	0.079	5.33
	Female RW 3	1, 188	0.165	0.685	0.29
	Female RW 4	1, 188	17.775	< 0.001	28.21
	Female RW 5	1, 188	2.271	0.133	3.90
Species × Centroid size	Female RW 1	1, 188	4.495	0.035	7.63
	Female RW 2	1, 188	3.593	0.060	6.12
	Female RW 3	1, 188	1.705	0.193	2.93
	Female RW 4	1, 188	3.084	0.081	5.27
	Female RW 5	1, 188	0.595	0.442	1.03
Species × Environmental PC 1	Female RW 1	1, 188	0.136	0.713	0.24
	Female RW 2	1, 188	10.500	0.001	17.28
	Female RW 3	1, 188	0.790	0.375	1.37
	Female RW 4	1, 188	0.170	0.681	0.29
	Female RW 5	1, 188	2.135	0.146	3.67
Species × Environmental PC 2	Female RW 1	1, 188	0.203	0.653	0.35
	Female RW 2	1, 188	0.537	0.464	0.93
	Female RW 3	1, 188	0.978	0.324	1.69
	Female RW 4	1, 188	0.073	0.787	0.13
	Female RW 5	1, 188	3.216	0.075	5.49
Species × Environmental PC 3	Female RW 1	1, 188	2.257	0.135	3.87
	Female RW 2	1, 188	3.891	0.050	6.62
	Female RW 3	1, 188	3.625	0.058	6.18
	Female RW 4	1, 188	2.785	0.097	4.77
	Female RW 5	1, 188	0.092	0.762	0.16

Table S7. Results from ANCOVAs on male life history variables using ‘species’ as a fixed factor, and ‘SL’ and environmental variables (PC 1 through 3) as covariates. All dependent variables were *z*-transformed.

Source	Dependent variable	d.f.	*F*	*p*	Relative variance explained (%)
Species	Male lean weight	1, 163	0.928	0.337	0.65
	Male fat content	1, 163	0.904	0.343	0.64
	GSI	1, 163	9.943	0.002	6.62
SL	Male lean weight	1, 163	1078.215	< 0.001	100.00
	Male fat content	1, 163	0.428	0.514	0.30
	GSI	1, 163	5.412	0.021	3.70
Environmental PC 1	Male lean weight	1, 163	0.019	0.890	0.01
	Male fat content	1, 163	40.941	< 0.001	23.11
	GSI	1, 163	0.667	0.415	0.47
Environmental PC 2	Male lean weight	1, 163	0.176	0.676	0.12
	Male fat content	1, 163	1.222	0.271	0.86
	GSI	1, 163	2.912	0.090	2.02
Environmental PC 3	Male lean weight	1, 163	0.028	0.868	0.02
	Male fat content	1, 163	10.640	0.001	7.05
	GSI	1, 163	1.764	0.186	1.23
Species * SL	Male lean weight	1, 163	2.026	0.157	1.41
	Male fat content	1, 163	0.615	0.434	0.43
	GSI	1, 163	0.008	0.930	0.01
Species × Environmental PC 1	Male lean weight	1, 163	0.821	0.366	0.58
	Male fat content	1, 163	28.991	< 0.001	17.38
	GSI	1, 163	3.322	0.070	2.30
Species × Environmental PC 2	Male lean weight	1, 163	0.272	0.603	0.19
	Male fat content	1, 163	13.516	< 0.001	8.81
	GSI	1, 163	0.529	0.468	0.37
Species × Environmental PC 3	Male lean weight	1, 163	0.165	0.685	0.12
	Male fat content	1, 163	9.158	0.003	6.12
	GSI	1, 163	0.038	0.845	0.03

Table S8. Results from ANCOVAs on female life history variables using ‘species’ as a fixed factor, and ‘SL’ and environmental variables (PC 1 through 3) as covariates. Embryo lean weight was corrected for embryo stage. All dependent variables were *z*-transformed.

Source	Dependent variable	d.f.	*F*	*p*	Relative variance explained (%)
Species	Female lean weight	1, 118	3.315	0.071	3.35
	Female fat content	1, 118	8.919	0.003	8.63
	Fecundity	1, 118	0.481	0.489	0.50
	Embryo fat content	1, 118	0.870	0.353	0.90
	Embryo lean weight	1, 118	0.622	0.432	0.64
	RA	1, 118	2.179	0.143	2.23
SL	Female lean weight	1, 118	518.554	< 0.001	100.00
	Female fat content	1, 118	7.152	0.009	7.01
	Fecundity	1, 118	22.452	< 0.001	19.62
	Embryo fat content	1, 118	0.010	0.920	0.01
	Embryo lean weight	1, 118	0.150	0.700	0.16
	RA	1, 118	0.820	0.367	0.85
Environmental PC 1	Female lean weight	1, 118	1.666	0.199	1.71
	Female fat content	1, 118	7.341	0.008	7.19
	Fecundity	1, 118	0.119	0.730	0.12
	Embryo fat content	1, 118	6.862	0.010	6.75
	Embryo lean weight	1, 118	0.445	0.506	0.46
	RA	1, 118	0.445	0.506	0.46
Environmental PC 2	Female lean weight	1, 118	0.000	0.990	0.00
	Female fat content	1, 118	23.701	< 0.001	20.53
	Fecundity	1, 118	21.828	< 0.001	19.16
	Embryo fat content	1, 118	0.357	0.551	0.37
	Embryo lean weight	1, 118	1.610	0.207	1.65
	RA	1, 118	15.448	0.000	14.21
Environmental PC 3	Female lean weight	1, 118	1.224	0.271	1.26
	Female fat content	1, 118	2.368	0.126	2.42
	Fecundity	1, 118	5.137	0.025	5.12
	Embryo fat content	1, 118	0.249	0.619	0.26
	Embryo lean weight	1, 118	0.062	0.803	0.06
	RA	1, 118	3.438	0.066	3.48
Species × SL	Female lean weight	1, 118	0.199	0.656	0.21
	Female fat content	1, 118	3.770	0.055	3.80
	Fecundity	1, 118	0.182	0.671	0.19
	Embryo fat content	1, 118	0.006	0.938	0.01
	Embryo lean weight	1, 118	0.289	0.592	0.30
	RA	1, 118	0.592	0.443	0.61
Species × Environmental PC 1	Female lean weight	1, 118	0.073	0.787	0.08
	Female fat content	1, 118	8.156	0.005	7.94
	Fecundity	1, 118	0.020	0.888	0.02
	Embryo fat content	1, 118	4.107	0.045	4.13
	Embryo lean weight	1, 118	0.548	0.461	0.57
	RA	1, 118	0.141	0.708	0.15
Species × Environmental PC 2	Female lean weight	1, 118	0.376	0.541	0.39
	Female fat content	1, 118	4.400	0.038	4.41
	Fecundity	1, 118	3.702	0.057	3.73
	Embryo fat content	1, 118	0.238	0.626	0.25
	Embryo lean weight	1, 118	1.007	0.318	1.04
	RA	1, 118	1.078	0.301	1.11
Species × Environmental PC 3	Female lean weight	1, 118	1.130	0.290	1.16
	Female fat content	1, 118	0.712	0.401	0.74
	Fecundity	1, 118	9.056	0.003	8.75
	Embryo fat content	1, 118	0.685	0.410	0.71
	Embryo lean weight	1, 118	0.374	0.542	0.39
	RA	1, 118	9.390	0.003	9.05

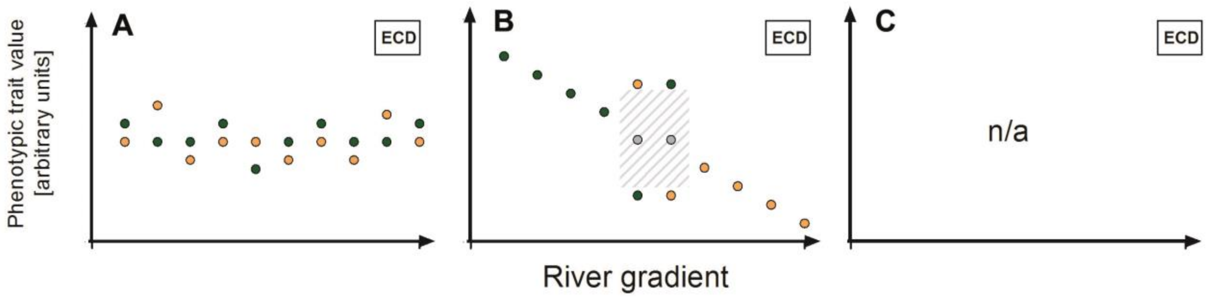

Figure S3. Appendix to Fig. 2 (see main text). Illustration of the potential outcome of ecological character displacement (ECD), where both species would diverge in opposing directions when (**A**) both species occur syntopically along the river gradient, or (**B**) in the overlap zone. Note that we depict non-directed ECD here, whereby both species vary unpredictably in both directions. If (**C**) certain components of the river gradient determine small-scale species distribution patterns and prevent syntopic occurrence of both species, ECD is not applicable.

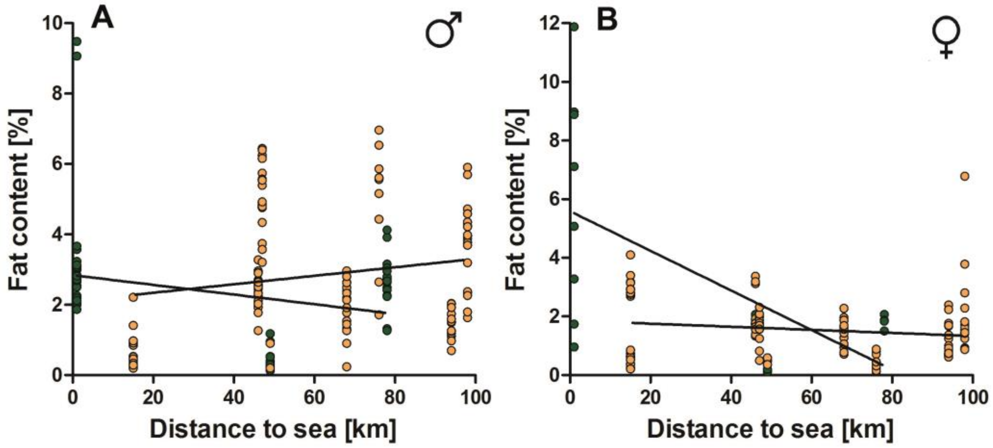

Figure S4. Relationship between somatic fat content [%] and distance to the sea [km] in (**A**) male and (**B**) female *Gambusia sexradiata* (orange) and *Gambusia yucatana* (green).

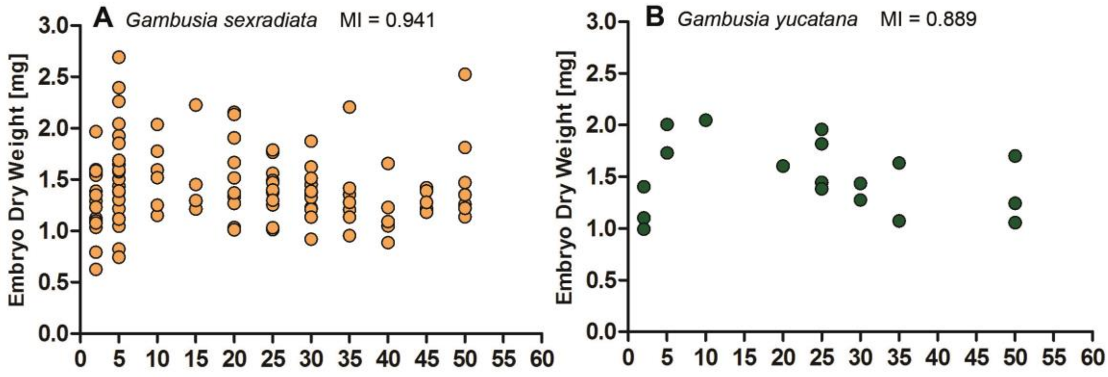

Figure S5. Scatterplots showing the relationships between mean embryo dry mass and stage of development in (**A**) *Gambusia sexradiata* (orange) and (**B**) *G. yucatana* (green). The matrotrophy index (MI) is the estimated dry mass at birth divided by dry mass at fertilization.

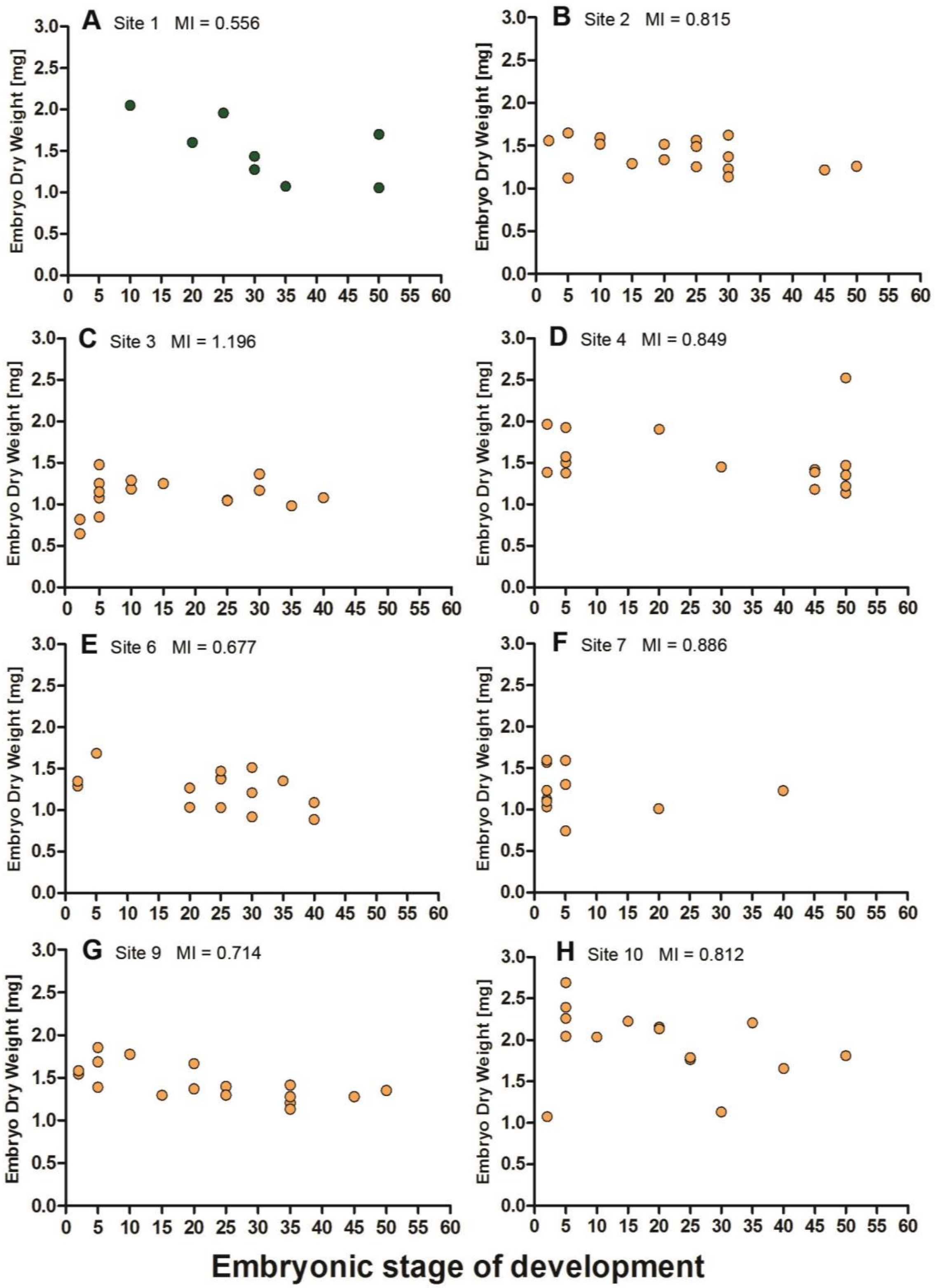

Figure S6. Scatterplots showing the relationships between mean embryo dry mass and stage of development in *Gambusia sexradiata* (orange) and *G. yucatana* (green) at different sampling sites. The matrotrophy index (MI) is the estimated dry mass at birth divided by dry mass at fertilization. The MI was calculated for each species and population separately if $n \geq 8$ pregnant females were available. Due to low sample sizes, sites 5 and 8 are not shown, nor were sufficient data available for *G. yucatana* from site 3.

Table S9. Results of principal component analysis of all male phenotypic traits (only *G. sexradiata*). Axis loadings ≥ |0.6| are highlighted in bold.

	PC 1	PC 2	PC 3	PC 4	PC 5
Male RW 1 (size corrected residuals)	**0.686**	0.105	0.474	0.353	0.121
Male RW 2 (size corrected residuals)	**0.725**	0.053	-0.226	-0.444	-0.146
Male RW 3 (size corrected residuals)	0.311	**-0.684**	-0.451	0.135	-0.055
Male RW 4 (size corrected residuals)	-0.100	-0.102	**0.923**	-0.019	-0.058
Male RW 5 (size corrected residuals)	0.001	-0.017	-0.007	0.003	**0.939**
Male lean weight (size corrected residuals)	0.122	**0.600**	-0.213	-0.167	0.383
SL	0.004	-0.009	-0.038	**0.942**	-0.041
Male fat content	-0.068	**0.754**	-0.098	0.131	-0.153
GSI	**0.692**	-0.201	-0.124	0.037	0.058

Table S10. Results of principal component analysis of all female phenotypic traits (only *G. sexradiata*). Axis loadings ≥ |0.6| are highlighted in bold.

	PC 1	PC 2	PC 3	PC 4	PC 5	PC 6
Female RW 1 (size corrected residuals)	0.369	**-0.711**	-0.005	-0.031	0.092	0.163
Female RW 2 (size corrected residuals)	0.129	0.030	**0.777**	0.087	-0.129	0.085
Female RW 3 (size corrected residuals)	0.219	0.228	-0.048	**0.826**	-0.097	0.052
Female RW 4 (size corrected residuals)	-0.014	**0.908**	0.020	-0.012	0.042	0.055
Female RW 5 (size corrected residuals)	0.356	0.047	-0.309	-0.247	0.389	0.090
Female lean weight (size corrected residuals)	0.016	0.458	-0.023	**-0.712**	-0.226	0.032
Fecundity (size corrected residuals)	**0.924**	-0.108	-0.168	0.079	0.001	0.012
Embryo lean weight (size corrected residuals)	-0.114	-0.004	**0.841**	-0.147	0.219	-0.065
Female fat ontent	-0.019	-0.034	0.127	0.084	**0.851**	-0.038
Embryo fat content	-0.146	0.108	0.244	0.031	-0.416	-0.545
RA	**0.838**	-0.201	0.323	0.204	0.045	0.005
SL	-0.038	0.005	0.123	0.035	-0.108	**0.874**

Table S11. Phenotypic trait divergence across *G. sexradiata* populations based on squared Euclidean distances. Males above, females below. Note that sites 1 and 8 did not harbour *G. sexradiata*.

	Pop. 2	Pop. 3	Pop. 4	Pop. 5	Pop. 6	Pop. 7	Pop. 9	Pop. 10
Pop. 2		6.764	8.028	1.877	0.884	4.711	4.788	2.739
Pop. 3	7.990		4.993	4.655	3.159	3.270	2.503	4.292
Pop. 4	4.681	6.093		6.825	5.038	1.857	4.042	5.125
Pop. 5	2.873	5.079	5.846		2.200	4.216	4.619	4.553
Pop. 6	1.959	2.828	4.836	2.753		2.430	2.388	1.275
Pop. 7	4.263	7.352	6.210	1.707	4.607		4.607	1.604
Pop. 9	5.376	4.307	5.037	2.319	5.171	4.007		4.116
Pop. 10	6.145	7.256	7.488	4.146	5.532	7.553	5.104	

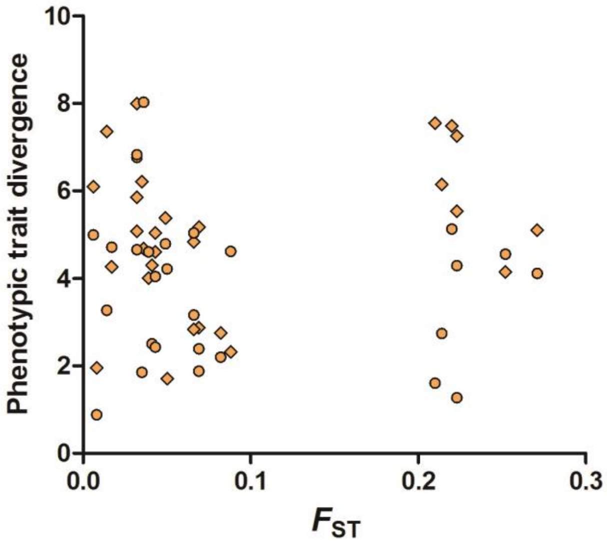

Figure S7. Scatterplot showing mean phenotypic trait divergence (Tab. S11) as a function of population genetic differentiation (estimated through pairwise F_{ST}) across populations of *Gambusia sexradiata*. A partial Mantel test found no correlation of phenotypic and genetic divergence in both sexes (males: r = -0.158, p = 0.474, circles; females: r = 0.278, p = 0.483, squares).
